# Complete Chloroplast Genomes of 14 Mangroves: Phylogenetic and Comparative Genomic Analyses

**DOI:** 10.1155/2020/8731857

**Published:** 2020-05-05

**Authors:** Chengcheng Shi, Kai Han, Liangwei Li, Inge Seim, Simon Ming-Yuen Lee, Xun Xu, Huanming Yang, Guangyi Fan, Xin Liu

**Affiliations:** ^1^School of Future Technology, University of Chinese Academy of Sciences, Beijing 101408, China; ^2^BGI-Qingdao, BGI-Shenzhen, Qingdao 266555, China; ^3^Integrative Biology Laboratory, Nanjing Normal University, Nanjing 210046, China; ^4^Comparative and Endocrine Biology Laboratory, Translational Research Institute-Institute of Health and Biomedical Innovation, School of Biomedical Sciences, Queensland University of Technology, Woolloongabba 4102, Australia; ^5^State Key Laboratory of Quality Research in Chinese Medicine, Institute of Chinese Medical Sciences, University of Macau, Macau, China; ^6^BGI-Shenzhen, Shenzhen 518083, China; ^7^China National GeneBank, BGI-Shenzhen, Shenzhen 518120, China; ^8^State Key Laboratory of Agricultural Genomics, BGI-Shenzhen, Shenzhen 518083, China

## Abstract

Mangroves are a group of plant species that occupy the coastal intertidal zone and are major components of this ecologically important ecosystem. Mangroves belong to about twenty diverse families. Here, we sequenced and assembled chloroplast genomes of 14 mangrove species from eight families spanning five rosid orders and one asterid order: Fabales (*Pongamia pinnata*), Lamiales (*Avicennia marina*), Malpighiales (*Excoecaria agallocha*, *Bruguiera sexangula*, *Kandelia obovata*, *Rhizophora stylosa*, and *Ceriops tagal*), Malvales (*Hibiscus tiliaceus*, *Heritiera littoralis*, and *Thespesia populnea*), Myrtales (*Laguncularia racemosa*, *Sonneratia ovata*, and *Pemphis acidula*), and Sapindales (*Xylocarpus moluccensis*). These chloroplast genomes range from 149 kb to 168 kb in length. A conserved structure of two inverted repeats (IRa and IRb, ~25.8 kb), one large single-copy region (LSC, ~89.0 kb), and one short single-copy region (SSC, ~18.9 kb) as well as ~130 genes (85 protein-coding, 37 tRNAs, and 8 rRNAs) was observed. We found the lowest divergence in the IR regions among the four regions. We also identified simple sequence repeats (SSRs), which were found to be variable in numbers. Most chloroplast genes are highly conserved, with only four genes under positive selection or relaxed pressure. Combined with publicly available chloroplast genomes, we carried out phylogenetic analysis and confirmed the previously reported phylogeny within rosids, including the positioning of obscure families in Malpighiales. Our study reports 14 mangrove chloroplast genomes and illustrates their genome features and evolution.

## 1. Introduction

Mangroves grow on the intertidal zone of the ocean, the transition zone connecting the land and ocean. Mangrove ecosystems provide essential habitats for marine creatures and benthic organisms and play important roles in regulating energy cycle and maintaining biodiversity [1, 2]. According to their habitats, root morphology, and salt metabolism patterns, mangroves are generally categorized into true mangroves and mangrove associates (or semi-mangroves) [3]. The true mangroves exclusively live in mangrove ecosystems and usually have distinct marine environment adaptations, including the ability to grow in seawater, complex root structures (allowing enhanced nutrient absorption and respiratory metabolism), and viviparous reproduction (seeds germinating on trees) [4]. Semi-mangroves are amphibious, and many can inhabit both terrestrial and aquatic environments (for instance, *Pongamia pinnata* (L.) Pierre). In mangrove ecosystems, they may grow at the edge of the true mangroves and are often dominant species on degraded beaches.

There are more than 80 mangrove species, covering approximately twenty families [5, 6]. Due to their ecological importance, wide distribution, and unique biological features for adaptation, the genome features and genome evolution of these species would be of considerable interest yet remain largely unexplored. As an essential organelle of plants, the chloroplast has an independent genome with stable sequence structure and a relatively conserved number of genes associated with energy production and metabolism. Chloroplast genes such as *rbcL* and *psbA* were once evidenced to be resultful in inferring the evolutionary origins and phylogenetic relationship of mangroves species from different clades or geographical regions [7–9]. DNA barcodes of *rbcL*, *matK*, and *trnH-psbA* genes have also been used to identify unknown mangrove species [6]. However, whole chloroplast genomes of mangrove species were limited until now [10]. Detailed whole chloroplast genome comparison and phylogenetic analysis has to date been lacking. In order to acquire more mangrove genetic resources and determine the evolutionary location of mangroves in rosids, we sequenced and assembled the complete chloroplast genomes of 14 mangrove species, including *Pongamia pinnata* (L.) Pierre, *Avicennia marina* (Forssk.) Vierh., *Excoecaria agallocha* L., *Bruguiera sexangula* (Lour.) Poir., *Kandelia obovata* Sheue, Liu & Yong, *Rhizophora stylosa* Griff., *Ceriops tagal* (Perr.) C.B.Rob., *Hibiscus tiliaceus* L., *Heritiera littoralis* Dryand., *Thespesia populnea* (L.) Sol. ex Correa, *Laguncularia racemosa* (L.) C.F. Gaertn., *Sonneratia ovata* Backer, *Pemphis acidula* Forst., and *Xylocarpus moluccensis* (Lamk.) Roem.. They represent mangroves of eight families, five rosid orders, and one asterid order (as an outgroup for the phylogenetic analysis). We examined their genome structures and gene contents. Comparative genomics and molecular evolution analyses were performed to illustrate mangrove chloroplast genome features further and reveal relationships among mangrove species.

## 2. Materials and Methods

### 2.1. Sequencing, Chloroplast Genome Assembly, and Annotation

Fresh leaves of mangroves were provided by collaborators in Guangzhou, China. DNA were extracted according to a CTAB method and then sequenced on a BGISEQ-500 platform. After sequencing, we randomly extracted five million pair-end reads. We used the MITObim v1.9 [11] for the initial assembly, following a closest reference-based strategy. The size of each chloroplast genome was estimated by SPAdes v3.13.0 [12]. With the initial assembly and the estimated genome size, we applied NOVOPlasty v2.7.2 [13] to assemble the complete chloroplast genome. Finally, we carried out manual curation to obtain circular sequences.

Chloroplast genes (including protein coding genes, rRNA genes, and tRNA genes) were predicted and annotated by GeSeq [14] with the MPI-MP chloroplast reference option. The identity cutoffs for protein and rRNA searching were set as 60 and 85, respectively. ARAGORN v1.2.38 [15] was used to annotate tRNAs. Genes were visualized using OGDRAW [16]. IR (inverted repeat) boundaries were identified by chloroplast genome self-alignment using BLAST v2.2.6 [17] (-p blastn -m 8 -F F -e 1). Regions aligned reversely and of the same length were manually curated as inverted repeat regions. Simple sequence repeats (SSRs) with 1-6 bp repeat units were detected using MISA v2.0 [18]. The minimum repeat times were set to be 10 for mononucleotides, 5 for dinucleotide, 4 for trinucleotide, 3 for tetranucleotide, 3 for pentanucleotide, and 3 for hexanucleotide.

### 2.2. Phylogenetic Analysis

A total of 44 conserved genes (*atpA*, *atpB*, *atpE*, *atpH*, *atpI*, *ccsA*, *cemA*, *matK*, *ndhA*, *ndhC*, *ndhG*, *ndhI*, *ndhJ*, *petA*, *petN*, *psaA*, *psaB*, *psaC*, *psaJ*, *psbA*, *psbC*, *psbD*, *psbE*, *psbF*, *psbH*, *psbI*, *psbJ*, *psbT*, *rbcL*, *rpoC1*, *rpoC2*, *rpl14*, *rpl2*, *rpoA*, *rpoB*, *rps11*, *rps14*, *rps15*, *rps19*, *rps2*, *rps3*, *rps4*, *rps8*, and *ycf3*) found in all 71 plant chloroplast genomes were used to construct robust phylogenetic trees (species listed in Table S2). The coding sequences were aligned by MAFFT v7.407 [19] with the “--auto --adjustdirection” setting. Based on the global alignments, the phylogenetic trees of the 71 representative species were constructed using several methods. Both partitioned and nonpartitioned strategies were implemented with different phylogenetic inference tools based on the concatenated aligned sequences from the 44 conserved genes, including (1) a BI tree constructed using MrBayes v3.2.7 [20] with a single priori GTR+GAMMA model, (2) a ML tree constructed using RAxML v8.2.12 [21] with a GTRGAMMA model, (3) a BI tree constructed using MrBayes with the best partition schemed models estimated by PartitionFinder v2.1.1 [22], and (4) a ML tree constructed using RAxML with the best partition schemed models estimated by PartitionFinder, as well as two trees using methods in (1) and (2) with four commonly used genes (*ndhF*, *matK*, *rbcL*, and *atpB*). For the Bayesian inference, MCMC analysis was run for 1,000,000 generations with four chains and sampling every 1,000 generations. The first 25% trees were discarded, and the final consensus tree was summarized using the remaining trees. For ML trees, the bootstrap number was set to 1,000. The trees were assessed using CONSEL v1.20 [23].

### 2.3. Ka/Ks Calculation

The nonsynonymous (Ka) and synonymous (Ks) substitution ratios (Ka/Ks) of genes in 14 mangroves, as well as in species from Lamiales, Fabales, Malpighiales, Malvales, Myrtales, Sapindales, Oxalidales, Celastrales, Fagales, Cucurbitales, Rosales, Brassicales, Huerteales, Geraniales, and Saxifragales (Table S2), were calculated. For all the species in an order, we selected one species outside the order to be used for comparison and Ka/Ks calculation. As these orders cover Asterid I, Rosid I, and Rosid II of core eudicots, we further chose one species from orders Asterales (*Helianthus divaricatus*: NC_023109.1), Zygophyllales (*Larrea tridentata*: NC_028023.1), Geraniales (*Hypseocharis bilobata*: NC_023260.1), and Vitales (*Vitis rotundifolia*: NC_023790.1) as outgroups of Asterid I, Rosid I, Rosid II, and the remaining rosid species. The two Lamiales species from asterids were compared to *Helianthus divaricatus* (NC_023109.1). Pairwise alignments were processed using MAFFT v7.407 [19], and Ka/Ks values were calculated using the KaKs Calculator [24]. Genes with low Ks values (cutoff 0.1, determined by considering the Ks distribution) were excluded as genes with unreliable omega values.

### 2.4. Synteny and Divergence Analyses

Genomic comparison and similarity calculations were performed using mVISTA [25]. The most closely related species used for synteny and divergence comparison for 14 mangroves were *Euphorbia tirucalli* (NC_042193.1), *Erythroxylum novogranatense* (NC_030601.1), *Wisteria floribunda* (NC_027677.1), *Gossypium lobatum* (NC_039569.1), *Hibiscus rosa-sinensis* (NC_042239.1), *Heritiera angustata* (NC_037784.1), *Xylocarpus granatum* (NC_039925.1), *Trapa natans* (NC_042895.1), *Punica granatum* (NC_035240.1), *Lumnitzera littorea* (NC_039752.1), and *Aphelandra knappiae* (NC_041424.1). For comparison within orders, the whole chloroplast genome sequences of Lamiales (*Sesamum indicum*: NC_016433.2, *Lindenbergia philippensis*: NC_022859.1, *Ajuga reptans*: NC_023102.1, *Hesperelaea palmeri*: NC_025787.1, *Scrophularia takesimensis*: NC_026202.1, *Tanaecium tetragonolobum*: NC_027955.1, *Erythranthe lutea*: NC_030212.1, *Paulownia coreana*: NC_031435.1, *Haberlea rhodopensis*: NC_031852.1, *Aloysia citrodora*: NC_034695.1, *Echinacanthus lofouensis*: NC_035876.1, and one mangrove *Avicennia marina*), Malpighiales (*Byrsonima coccolobifolia*: NC_037191.1, *Erythroxylum novogranatense*: NC_030601.1, *Garcinia mangostana*: NC_036341.1, *Hirtella racemosa*: NC_024060.1, *Ricinus communis*: NC_016736.1, *Salix interior*: NC_024681.1, *Viola seoulensis*: NC_026986.1, and mangroves *Bruguiera sexangula*, *Ceriops tagal*, *Excoecaria agallocha*, *Kandelia obovata*, and *Rhizophora stylosa*), Myrtales (*Allomaieta villosa*: NC_031875.1, *Eucalyptus obliqua*: NC_022378.1, *Lagerstroemia fauriei*: NC_029808.1, and mangroves *Laguncularia racemosa*, *Pemphis acidula*, and *Sonneratia ovata*), Sapindales (*Azadirachta indica*: NC_023792.1, *Boswellia sacra*: NC_029420.1, *Citrus aurantiifolia*: NC_024929.1, *Leitneria floridana*: NC_030482.1, *Sapindus mukorossi*: NC_025554.1, *Spondias bahiensis*: NC_030526.1, and mangrove *Xylocarpus moluccensis*), and Malvales species (*Gossypium arboreum*: NC_016712.1, *Daphne kiusiana*: NC_035896.1, and mangroves *Hibiscus tiliaceus* and *Thespesia populnea*) were aligned with MAFFT (v7.407) [19], and single nucleotide polymorphisms (SNPs) and insertion and deletions (InDels) were identified and counted in 200 bp windows with an in-house python script.

## 3. Results and Discussions

### 3.1. Chloroplast Genome Features

Using a reference genome-based strategy (see Materials and Methods), a total of 483 Mb chloroplast data were generated for 14 mangrove species in six orders: Fabales (*Pongamia pinnata*), Lamiales (*Avicennia marina*), Malpighiales (*Excoecaria agallocha*, *Bruguiera sexangula*, *Kandelia obovata*, *Rhizophora stylosa*, and *Ceriops tagal*), Malvales (*Hibiscus tiliaceus*, *Heritiera littoralis*, and *Thespesia populnea*), Myrtales (*Laguncularia racemosa*, *Sonneratia ovata*, and *Pemphis acidula*), and Sapindales (*Xylocarpus moluccensis*). The coverage of chloroplasts ranges from 28X to 526X (Table 1), which might be related with different chloroplast DNA content in the total DNA. The chloroplast genomes were assembled into single circular sequences, ranging from 149 kb (*Pongamia pinnata*) to 168 kb (*Kandelia obovata*) (Table 1 and Figure S1). We observed typical quadripartite structures in these chloroplast genomes, with two inverted repeat regions (IRa and IRb), a short single-copy (SSC) region, and a long single-copy (LSC) region. The lengths of these four regions are similar among species from the same order but slightly different between orders. The average lengths of IRs of mangrove species in Fabales, Lamiales, Malpighiales, Malvales, Myrtales, and Sapindales are approximately 23.6 kb, 25.6 kb, 26.3 kb, 26.0 kb, 25.3 kb, and 27.0 kb, respectively. The sizes of the SSC range from 17.9 kb to 20.0 kb and the LSC range from 83 kb to 91 kb (Table 1). We found a GC content between 35% and 39% in the 14 chloroplast genomes. The GC content of IR regions (~43%) is higher than those of the SSC (30%) and LSC (34%) regions (Table S1).

The number of chloroplast genes is usually conserved [26], with subtle differences between different species [27, 28]. The mangrove chloroplasts contain ~85 protein-coding genes, ~37 tRNA genes, and eight rRNA genes (Table 2). The gene components of photosystem I (five genes), cytochrome b/f complex (six genes), ATP synthase (six genes), NADH dehydrogenase (12 genes), Rubisco large subunit (*rbcL*), RNA polymerase (four genes), assembly/stability of photosystem I (*ycf3* and *ycf4*), RNA processing (*matK*), chloroplast envelope membrane protein (*cemA*), cytochrome c synthesis (*ccsA*), ATP-dependent protease (*clpP*), fatty acid biosynthetic (*accD*), and proteasome subunit beta type-1 (*pbf1*) are the same in all the mangrove chloroplasts. We only found *infA*, a chloroplast genome translation initiation factor gene, in *Avicennia marina* and *Heritiera littoralis* (Table 2). This agrees with the fact that *infA* is commonly lost in angiosperms, especially in rosid species [29].

### 3.2. Simple Sequence Repeat Content

Simple sequence repeats (SSRs) are tandem repeats (1~6 bp units repeated multiple times) in the genome which have been widely applied as markers for population studies and crop improvements [30–33]. In this study, we detected and compared SSRs in the mangrove and 57 terrestrial plant chloroplast genomes (Table S2). The SSR contents are highly variable among species (Figure S2). Of the 14 mangroves, *Kandelia obovate* has the highest number of SSRs (194), while *Avicennia marina* has the fewest (61) (Table 3). Comparing between orders, the Malpighiales (number of SSRs ranges from 133 to 194; *Excoecaria agallocha*, *Bruguiera sexangula*, *Kandelia obovata*, *Ceriops tagal*, and *Rhizophora stylosa*) has more SSRs than species of orders Malvales (number of SSRs ranges from 80 to 110; *Hibiscus tiliaceus*, *Heritiera littoralis*, and *Thespesia populnea*) and Myrtales (number of SSRs ranges from 88 to 118; *Laguncularia racemosa*, *Sonneratia ovata*, and *Pemphis acidula*) (Table 3). Assessing SSR categories in the 14 mangrove chloroplast genomes, we found that the mononucleotide type accounted for at least half of the total SSRs (in *Laguncularia racemosa* and *Avicennia marina* up to 80%). A/T tandem repeats are most frequent, followed by dinucleotide, tetranucleotide, trinucleotide, pentanucleotide, and hexanucleotide repeats. Similar patterns of SSR variability and constitution were also observed in the 57 terrestrial plant chloroplasts (Figure S2). We propose that the SSRs identified here can serve as useful genetic resources for future population and evolution studies.

### 3.3. Phylogenetic Relationships of Mangroves

**Figure 1:**
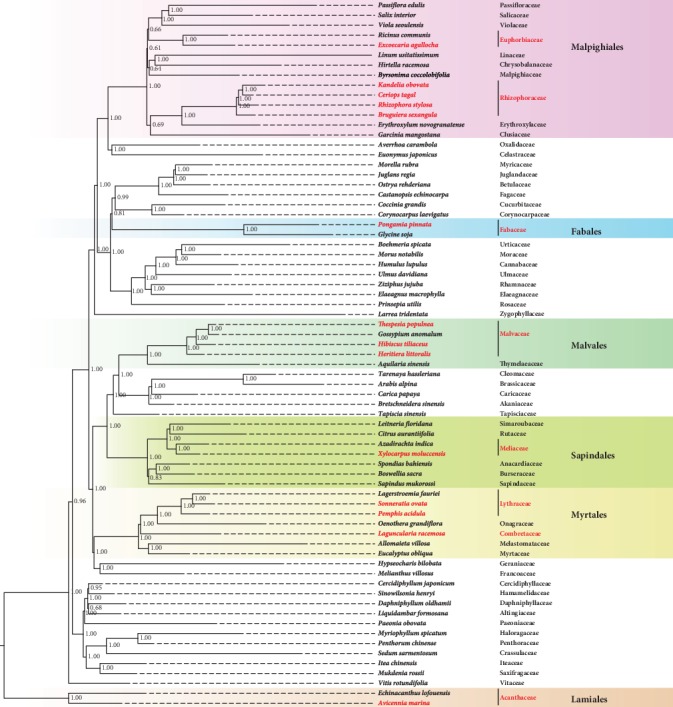
The Bayesian-inferred phylogenetic tree based on all chloroplast genes of 14 mangroves and 57 land plant species. Mangroves are indicated in red, and the background colors highlight the orders of mangroves and their sister species. Posterior probabilities are indicated near the branches.

Similar to mitochondrial genomes used in vertebrate genetics, chloroplast genomes are widely used to settle phylogenetic and evolutionary disputes [28]. In order to reveal the phylogenetic relationships of mangroves, we constructed phylogenetic trees from chloroplast genomes from the 14 mangroves species and 57 terrestrial plant families in 16 rosid orders and one asterid order (a data set of 71 plant species) (Table S2). Based on these complete chloroplast genomes, we identified 44 highly conserved genes in the 71 species and constructed three Bayesian inference (BI) trees and three maximum-likelihood (ML) trees (see Materials and Methods; Figure 1 and Figures S3-S7). Three trees constructed using BI and ML strategies exhibited the same topology (Figure 1, Figure S3, Figure S5, and Table S3). Thus, we have produced a well-supported phylogenetic tree of mangrove and terrestrial plants.

Based on our phylogenetic tree, we found species from the same order to be in one group, and Rosid I (Malpighiales, Oxalidales, Celastrales, Fagales, Cucurbitales, Fabales, Rosales, and Zygophyllales), Rosid II (Malvales, Brassicales, Huerteales, Sapindales, Myrtales, and Geraniales), and a clade of rosids (Saxifragales and Vitales) were classified. For mangrove species, *Avicennia marina* is close to the other asterid terrestrial plant *Echinacanthus lofouensis*. Using these two species as outgroups, we obtained a clear phylogenetic relationship of the rest 13 rosid mangrove species. Myrtales is close to Geraniales in this tree and contains five families, of which Myrtaceae and Melastomataceae are in one clade, while Onagraceae and Lythraceae (including two mangroves *Sonneratia ovata* and *Pemphis acidula*) are in another clade. The relationships indicated here are consistent with a reported ML tree of Myrtales species [34]. Furthermore, we found that Combretaceae (*Laguncularia racemosa*) is a separate node close to Onagraceae and Lythraceae, supported by 1.00 posterior probability. For Sapindales, there is one mangrove *Xylocarpus moluccensis*, a member of Meliaceae whose position coincides with a previous study [35]. For Malvales, three mangroves (*Hibiscus tiliaceus*, *Heritiera littoralis*, and *Thespesia populnea*) together with Huerteales and Brassicales species are clustered as neighboring orders. The relationship of genera within the family of Malvaceae is in agreement with trees in *Aquilaria sinensis* [36] and *Heritiera angustata* [37] chloroplast studies, and we further confirmed that the semi-mangrove *Thespesia populnea* is close to *Gossypium* species. However, for Malpighiales, an order of high morphological and ecological diversities, the phylogenetic relationship of different families especially Linaceae was less resolved. Other than the grouping of families Linaceae and Euphorbiaceae, our phylogenetic tree is consistent with a previous study which employed 82 plastid genes of 58 species from Malpighiales [38]. We found that Euphorbiaceae constitutes a single branch, while Rhizophoraceae (including four mangroves *Bruguiera sexangula*, *Kandelia obovata*, *Rhizophora stylosa*, and *Ceriops tagal*) is a neighboring branch to Erythroxylaceae and Clusiaceae. Also, Linaceae forms a sister lineage with Chrysobalanaceae and Malpighiaceae. Again, these relationships are largely accordant with a study of *Linum* plastome [39]. Finally, our phylogenetic tree supports a sister relationship between the mangrove *Pongamia pinnata* with the other orders of Rosales, Cucurbitales, and Fagales.

### 3.4. Synteny and Divergence of the Chloroplast Genomes

**Figure 2:**
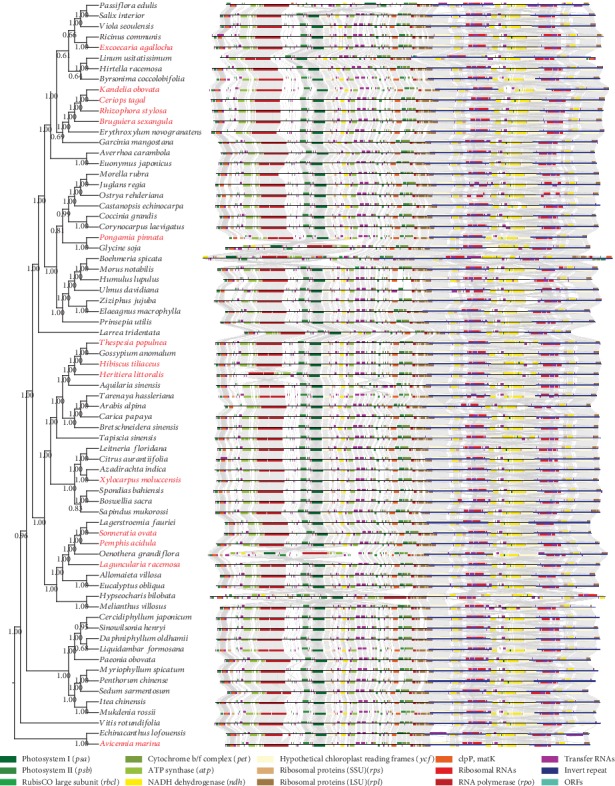
Synteny between the chloroplast genomes. The species are shown in the phylogenetic tree with red fonts to indicate the mangrove species, and the synteny is shown by linking the homologous genes. Colors at the bottom indicate genes with different functions.

**Figure 3:**
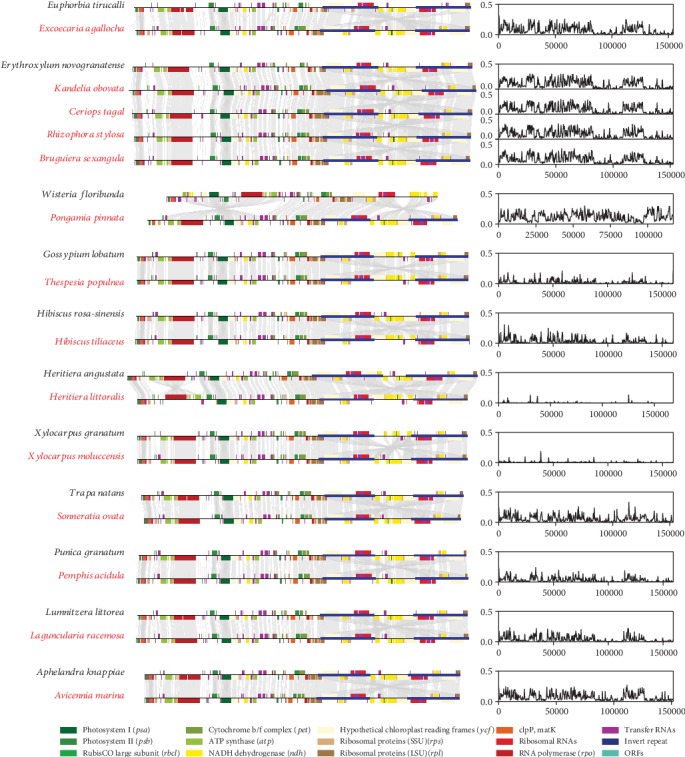
Pairwise comparisons of mangrove species and their most closely related species. Whole chloroplast genome comparisons are shown on the left, and the corresponding divergences are shown on the right.

**Figure 4:**
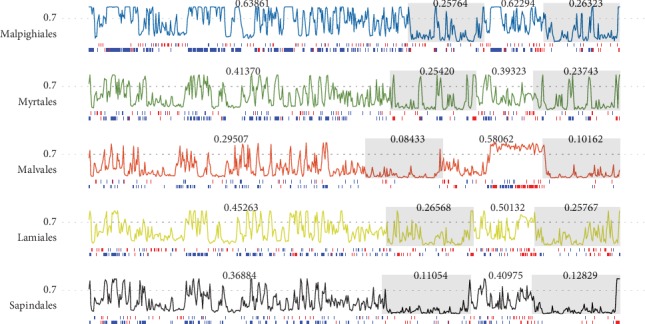
Divergence within orders of Malpighiales, Myrtales, Malvales, Lamiales, and Sapindales. The IR regions are shown in grey background, and the average divergences of each region are indicated above the lines in the corresponding regions. Two bars at the bottom show the SNPs and InDels in both the gene regions (red) and the intergenic regions (blue).

We next analyzed the synteny and divergence between the mangrove and related chloroplast genomes. For mangrove species, the genomes have a conserved gene order similar to sister clades, except *Heritiera littoralis* which probably had been subjected to segmental rearrangements (Figures 2 and 3). Compared to its closely related species *Hibiscus tiliaceus* and *Thespesia populnea*, we found a notable rearrangement at position 8,109 bp to 33,498 bp in the *Heritiera littoralis* chloroplast genome. This region encodes 16 genes, including *trnC-GCA*, *petN*, *psbM*, *trnD-GUC*, *trnY-GUA*, *trnE-UUC*, *rpoB*, *rpoC1*, *rpoC2*, *rps2*, *atpI*, *atpH*, *atpF*, *atpA*, *trnR-UCU*, and *trnS-CGA*. Assessing the genetic divergence of mangrove chloroplast genomes by the most closely related species and within the orders (see Materials and Methods), we found the lowest divergence in the genera *Heritiera* and *Xylocarpus* (Figure 3). Compared to *Heritiera* and *Xylocarpus*, there is a relatively higher divergence between *Hibiscus rosa-sinensis* and *Hibiscus tiliaceus*, reflecting a higher level of genetic polymorphism of chloroplast genomes within genus *Hibiscus*. We also observed a more distinct divergence between species from one family/order in most other comparisons (Euphorbiaceae: *Euphorbia tirucalli* vs. *Excoecaria agallocha*, Malpighiales: *Erythroxylum novogranatense* vs. *Kandelia obovata/Ceriops tagal/Rhizophora stylosa/Bruguiera sexangula*, Fabaceae: *Wisteria floribunda* vs. *Pongamia pinnata*, Malvaceae: *Gossypium lobatum* vs. *Thespesia populnea*, Lythraceae: *Trapa natans* vs. *Sonneratia ovata*, Lythraceae: *Punica granatum* vs. *Pemphis acidula*, Combretaceae: *Lumnitzera littorea* vs. *Laguncularia racemosa*, Acanthaceae: *Aphelandra knappiae* vs. *Avicennia marina*) (Figure 3). Furthermore, according to the whole genomic comparison among multiple chloroplasts within orders, we found that the similarity of coding regions is generally higher than that of the intergenic regions, and the tRNA and rRNA genes are almost identical in all species (Figure S8). For the four regions, variations in SSC and LSC regions are more frequent comparing to the IR regions, indicating the IRs to be more conserved than the single-copy sequences (Figure 4). This is consistent with reports on other plant chloroplasts [40–42].

### 3.5. Genes under Selective Pressures

**Figure 5:**
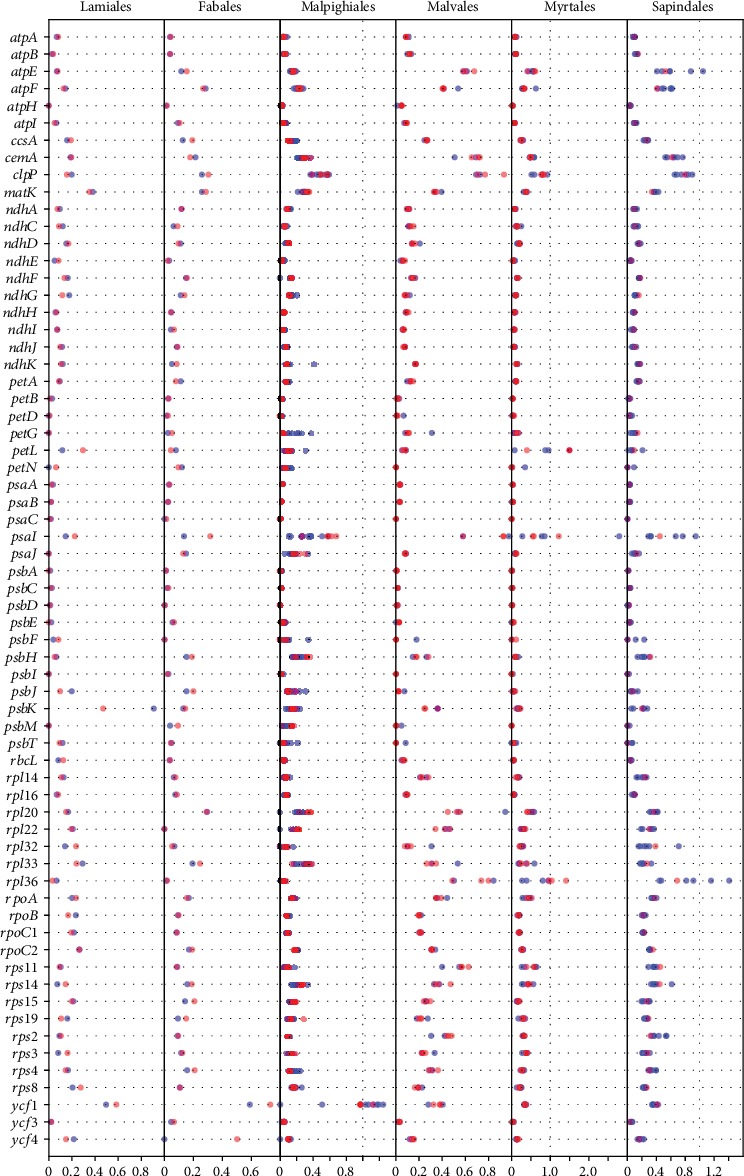
Ka/Ks values of protein-coding genes in chloroplasts. Ka/Ks values (horizontal axis) of genes in the mangrove species and their related species in six orders (Lamiales, Fabales, Malpighiales, Malvales, Myrtales, and Sapindales) are indicated. Red dots indicate Ka/Ks values of genes in mangrove species, while blue dots indicate Ka/Ks values in terrestrial species.

Genes in the chloroplasts are functionally important and might have been under selection during evolution. To analyze the selective pressures in mangrove chloroplast genomes, we calculated the nonsynonymous substitutions and synonymous substitution ratio (Ka/Ks) of coding genes in 14 mangrove species and 57 terrestrial plants (see Materials and Methods). Genes with Ka/Ks values above 1.0 should be under positive selection and might be candidate genes responsible for functional adaptations, while genes with values lower than 1.0 should be under negative (purifying) selection [43]. We found the Ka/Ks values of most gene pairs (within and between orders) to be lower than 1.0 (Figure 5), reflecting selection pressures to maintain the gene functions. For instance, genes involved in photosystems (*psaA*, *psaB*, *psaC*, *psbA*, *psbC*, *psbD*, *psbE*, *psbI*, and *psbM*), the cytochrome b/f complex (*petB*, *petD*, and *petG*), and some ATP synthases (*atpB* and *atpH*) in all species have Ka/Ks values close to 0. Genes encoding other ATP synthases (*atpA*, *atpE*, and *atpF*), NADH dehydrogenases (*ndhA*, *ndhC*, *ndhD*, *ndhE*, *ndhF*, *ndhG*, *ndhH*, *ndhI*, *ndhJ*, and *ndhK*), ribosomal proteins (*rps2*, *rps3*, *rps4*, *rps8*, *rps11*, *rps14*, *rps15*, and *rps19*), and RNA polymerases (*rpoA*, *rpoB*, *rpoC1*, and *rpoC2*) also have low Ka/Ks values (mostly between 0 and 0.5). Similar to other plants [39, 44], chloroplast genes involved in photosynthesis and energy metabolism are conserved (very low Ka/Ks ratios) in mangroves.

We further investigated the genes under positive selection. We found that the Ka/Ks values of four genes (*petL*, *psaI*, *rpl36*, and *ycf1*) were greater than 1.0 in the mangrove species *Excoecaria agallocha*, *Laguncularia racemosa*, *Pemphis acidula*, and *Sonneratia ovata*. These genes are from four different functional groups, including subunits of cytochrome (*petL*), subunits of photosystems (*psaI*), subunits of ribosomes (*rpl36*), and unclassified genes (*ycf1*). The gene *petL* is a component of the cytochrome b6/f complex required for photosynthesis. The Ka/Ks of *petL* is ~1.5 in mangrove species *Sonneratia ovata* and *Pemphis acidula*, and positive selection on this gene was found only in these two species (Figure 5 and Figures S9 and S10). For *psaI*, a member of photosystem I (PSI), we observed a Ka/Ks value of 1.22 in *Laguncularia racemosa* (family Combretaceae, order Myrtales), suggesting potential positive selection on this gene in this mangrove. Among all species, Ka/Ks values of *psaI* range widely (from 0.2 to 1.5, especially in Malpighiales, Myrtales, and Sapindales), which may reflect the different selection pressures and adaptations in the diverse clades examined. Although a report on tobacco showed a role for *psaI* in stabilizing PSI during leaf senescence [45], the function of the protein remains unknown in most plant species. For *rpl36* (LSU), the Ka/Ks values are greater than 1.0 in *Pemphis acidula* (1.41) and *Sonneratia ovata* (1.04). The loss of *rpl36* might result in severe morphological aberrations, low translational efficiency, and poor photoautotrophic growth [46]. We speculate that relaxed selection on this gene might be associated with adaptations of plants to highly diverse environments. Gene *ycf1* is one of the largest genes in the chloroplast genome, and there are usually two [39, 41] or one functional gene copies (i.e., one copy has become a pseudogene) [36, 44, 47, 48]. In this study, one functional copy and one fragment of the *ycf1* gene were annotated in 11 mangroves (*Avicennia marina*, *Xylocarpus moluccensis*, *Hibiscus tiliaceus*, *Excoecaria agallocha*, *Bruguiera sexangula*, *Kandelia obovata*, *Rhizophora stylosa*, *Ceriops tagal*, *Sonneratia ovata*, *Pemphis acidula*, and *Laguncularia racemosa*). Furthermore, we found that *ycf1* genes in many species from Rosid I (such as Malpighiales including mangrove *Excoecaria agallocha*) have Ka/Ks values around or higher than 1.0, while *ycf1* genes in species from Rosid II plants have lower Ka/Ks values (~0.4 in Malvales, Myrtales, Sapindales, Huerteales, and Brassicales). Together with the fact that the *ycf1* gene showed relatively lower sequence similarity among different species (Figure S8), we found different selection pressures on this gene in Rosid I and Rosid II here (Figure 5 and Figures S9 and S10). The observed potential positive selection on *petL*, *psaI*, and *rpl36* in mangroves (especially in the three mangroves *Laguncularia racemosa*, *Pemphis acidula*, and *Sonneratia ovata* of order Myrtales) and a relaxed selection pressure on *ycf1* possibly reflect the functional importance of those genes during adaptation.

## 4. Conclusions

Our study reports 14 complete mangrove chloroplast genomes, as well as a comprehensive comparative chloroplast genome analysis of mangrove and related plant species. The sequenced mangroves span six orders (five rosids and one asterid), making it the first large-scale study on mangrove chloroplast genomes. We found that mangrove chloroplast genomes are similar in structure and gene content. Notable exceptions include the retainment of the translation initiation factor gene *infA* in two mangrove species (the asterid *Avicennia marina* and the rosid *Heritiera littoralis*) and an inversion in the LSC region of mangrove *Heritiera littoralis*. We used our new mangrove genomes to create a well-supported phylogeny. Protein-coding genes of mangroves were found to be under pressure to maintain gene function, with only a small number of genes in a handful of species showing evidence of positive or relaxed selection. In conclusion, we report 14 complete chloroplast genomes from diverse mangrove species and analyzed their phylogeny and genome features. This study provides a useful resource for future studies on the evolution of mangroves and environmental adaptation.

## Figures and Tables

**Table 1:** Chloroplast genome features of the 14 mangroves species.

Species	Family	Extracted chloroplast data (Mb)	Mean coverage (×)	Total length (bp)	IR length (bp)	SSC length (bp)	LSC length (bp)
Fabales							
*Pongamia pinnata*	Fabaceae	4.30	28.72	149,635	23,653	18,534	83,795
Lamiales							
*Avicennia marina*	Acanthaceae	32.54	213.66	152,288	25,638	17,924	83,088
Malpighiales							
*Excoecaria agallocha*	Euphorbiaceae	11.82	73.10	161,667	26,525	19,336	89,282
*Bruguiera sexangula*	Rhizophoraceae	32.86	202.52	162,282	26,403	18,144	91,332
*Kandelia obovata*	Rhizophoraceae	88.42	526.82	168,008	26,320	19,955	95,413
*Rhizophora stylosa*	Rhizophoraceae	71.24	436.76	163,101	26,325	19,243	91,208
*Ceriops tagal*	Rhizophoraceae	42.89	260.80	164,476	26,313	19,153	92,697
Malvales							
*Hibiscus tiliaceus*	Malvaceae	18.75	116.25	161,318	26,159	19,717	89,283
*Heritiera littoralis*	Malvaceae	16.40	102.87	159,401	26,261	19,002	87,877
*Thespesia populnea*	Malvaceae	6.42	40.04	160,451	25,582	20,306	88,981
Myrtales							
*Pemphis acidula*	Lythraceae	35.84	223.93	160,051	25,695	18,886	89,775
*Sonneratia ovata*	Lythraceae	68.08	444.79	153,057	23,906	18,007	87,238
*Laguncularia racemosa*	Combretaceae	27.43	170.77	160,672	26,353	18,886	89,071
Sapindales							
*Xylocarpus moluccensis*	Meliaceae	25.98	163.08	159,317	27,000	17,998	87,319

**Table 2:** Gene content of the 14 mangrove chloroplast genomes.

	*Pongamia pinnata*	*Avicennia marina*	*Excoecaria agallocha*	*Bruguiera sexangula*	*Kandelia obovata*	*Rhizophora stylosa*	*Hibiscus tiliaceus*	*Heritiera littoralis*	*Thespesia populnea*	*Ceriops tagal*	*Laguncularia racemosa*	*Sonneratia ovata*	*Pemphis acidula*	*Xylocarpus moluccensis*
Total gene number	127	133	132	128	129	129	130	129	129	130	130	130	131	131
Transfer RNAs (tRNA)	35	37	37	37	37	37	37	36	37	37	37	37	37	37
Ribosomal RNAs (rRNAs)	8	8	8	8	8	8	8	8	8	8	8	8	8	8
Coding genes	84	88	87	83	84	84	85	85	84	85	85	85	86	86
Photosystems I	5	5	5	5	5	5	5	5	5	5	5	5	5	5
Photosystems II	15	14	14	14	14	14	14	14	14	14	14	14	14	14
Cytochrome b/f complex	6	6	6	6	6	6	6	6	6	6	6	6	6	6
ATP synthase	6	6	6	6	6	6	6	6	6	6	6	6	6	6
NADH dehydrogenase	12	12	12	12	12	12	12	12	12	12	12	12	12	12
Rubisco large subunit	1	1	1	1	1	1	1	1	1	1	1	1	1	1
RNA polymerase	4	4	4	4	4	4	4	4	4	4	4	4	4	4
Ribosomal proteins (small subunit)	14	14	14	13	13	13	14	14	14	14	14	14	15	15
Ribosomal proteins (large subunit)	10	11	11	10	11	11	11	11	11	11	11	11	11	11
Assembly/stability of photosystem I	2	2	2	2	2	2	2	2	2	2	2	2	2	2
RNA processing	1	1	1	1	1	1	1	1	1	1	1	1	1	1
Chloroplast envelope membrane protein	1	1	1	1	1	1	1	1	1	1	1	1	1	1
Cytochrome c synthesis	1	1	1	1	1	1	1	1	1	1	1	1	1	1
Proteins of unknown function	3	4	4	4	4	4	4	3	3	4	4	4	4	4
ATP-dependent protease	1	1	1	1	1	1	1	1	1	1	1	1	1	1
Fatty acid biosynthetic	1	1	1	1	1	1	1	1	1	1	1	1	1	1
Translation initiation factor	0	1	0	0	0	0	0	1	0	0	0	0	0	0
Putative uncharacterized protein	0	2	2	0	0	0	0	0	0	0	0	0	0	0
Proteasome subunit beta type-1	1	1	1	1	1	1	1	1	1	1	1	1	1	1

**Table 3:** Number of SSRs in the 14 mangrove chloroplast genomes. Mononucleotide, dinucleotide, trinucleotide, tetranucleotide, pentanucleotide, and hexanucleotide repeat units are abbreviated as u1 to u6.

Species	Total	u1	u1 : A/T	u1 : C/G	u2	u3	u4	u5	u6
*Pongamia pinnata*	130	84	82	2	31	6	7	1	1
*Avicennia marina*	61	49	45	4	1	4	7	0	0
*Excoecaria agallocha*	133	92	87	5	19	7	13	1	1
*Bruguiera sexangula*	175	106	103	3	31	14	18	5	1
*Kandelia obovata*	194	106	106	0	36	20	20	10	2
*Rhizophora stylosa*	169	115	111	4	22	13	8	9	2
*Ceriops tagal*	142	78	75	3	16	20	23	4	1
*Hibiscus tiliaceus*	86	55	53	2	15	4	10	2	0
*Heritiera littoralis*	110	76	74	2	7	6	13	6	2
*Thespesia populnea*	80	47	44	3	17	3	7	5	1
*Sonneratia ovata*	112	77	72	5	15	5	10	4	1
*Pemphis acidula*	88	64	64	0	7	7	9	1	0
*Laguncularia racemosa*	118	98	96	2	6	5	8	1	0
*Xylocarpus moluccensis*	98	72	71	1	6	6	11	3	0

## Data Availability

The 14 assembled chloroplast genome sequences along with the annotation can be found in CNSA (https://db.cngb.org/cnsa/) of CNGBdb under the accession number CNP0000567.

## Supplementary Materials

Supplementary Materials Figure S1: the whole chloroplast genomes of 14 mangroves. The inner circle marks the LSC, SSC, and IR regions. Genes' position and orientations are shown along the outer circle. Genes with different functions are colored. Figure S2: the SSR distribution in the 14 mangroves and 57 terrestrial plant chloroplast genomes. (A) Number of different SSR types in each species. Mangroves are marked in red. (B) The SSR numbers in 17 orders (dots for each species and bars for average number). Figure S3: the ML phylogenetic tree based on whole chloroplast genes of 14 mangroves and 57 land species. Figure S4: the BI phylogenetic tree based on whole chloroplast genes of 14 mangroves and 57 land species using partition model. Figure S5: the ML phylogenetic tree based on whole chloroplast genes of 14 mangroves and 57 land species using partition model. Figure S6: the BI phylogenetic tree based on four conserved genes (*ndhF*, *matK*, *rbcL*, and *atpB*) of 14 mangroves and 57 land species. Figure S7: the ML phylogenetic tree based on four conserved genes (*ndhF*, *matK*, *rbcL*, and *atpB*) of 14 mangroves and 57 land species. Figure S8: the genomic comparison and similarities of whole chloroplast sequences among mangroves and their related species within orders Lamiales, Fabales, Malpighiales, Malvales, Myrtales, and Sapindales. CNS: conserved noncoding sequence. Figure S9: the Ka/Ks values of chloroplast protein-coding genes in species from Oxalidales, Celastrales, Fagales, Cucurbitales, and Rosales (Rosid I clades). Figure S10: the Ka/Ks values of chloroplast protein-coding genes in species from Brassicales (Rosid II), Huerteales (Rosid II), Geraniales, and Saxifragales. Table S1: GC content in the 14 mangrove chloroplast genomes. Table S2: the 57 public terrestrial species used for genomic comparison analyses (SSR, phylogeny, and evolution). Table S3: the assessment and comparison of phylogenetic trees.

## References

[1] Serafy J. E., Shideler G. S., Araújo R. J., Nagelkerken I. (2015). Mangroves enhance reef fish abundance at the Caribbean regional scale. *PLoS One*.

[2] Mumby P. J., Edwards A. J., Arias-González J. E. (2004). Mangroves enhance the biomass of coral reef fish communities in the Caribbean. *Nature*.

[3] Tomlinson P. B. (1986). *The Botany of Mangroves*.

[4] Ball M. C. (1988). Ecophysiology of mangroves. *Trees*.

[5] Polidoro B. A., Carpenter K. E., Collins L. (2010). The loss of species: mangrove extinction risk and geographic areas of global concern. *PLoS One*.

[6] Wu F., Li M., Liao B., Shi X., Xu Y. (2019). DNA barcoding analysis and phylogenetic relation of mangroves in Guangdong Province China. *Forests*.

[7] Shi S., Huang Y., Zeng K. (2005). Molecular phylogenetic analysis of mangroves: independent evolutionary origins of vivipary and salt secretion. *Molecular Phylogenetics and Evolution*.

[8] Li X., Duke N. C., Yang Y. (2016). Re-evaluation of phylogenetic relationships among species of the mangrove genus *Avicennia* from Indo-West Pacific based on multilocus analyses. *PLoS One*.

[9] Lo E. Y. Y., Duke N. C., Sun M. (2014). Phylogeographic pattern of Rhizophora (Rhizophoraceae) reveals the importance of both vicariance and long-distance oceanic dispersal to modern mangrove distribution. *BMC Evolutionary Biology*.

[10] Yang Y., Zhang Y., Chen Y. (2019). Complete chloroplast genome sequence of the mangrove speciesKandelia obovata and comparative analyses with related species. *PeerJ*.

[11] Hahn C., Bachmann L., Chevreux B. (2013). Reconstructing mitochondrial genomes directly from genomic next-generation sequencing reads--a baiting and iterative mapping approach. *Nucleic Acids Research*.

[12] Bankevich A., Nurk S., Antipov D. (2012). SPAdes: a new genome assembly algorithm and its applications to single-cell sequencing. *Journal of Computational Biology*.

[13] Dierckxsens N., Mardulyn P., Smits G. (2017). NOVOPlasty: de novo assembly of organelle genomes from whole genome data. *Nucleic Acids Research*.

[14] Tillich M., Lehwark P., Pellizzer T. (2017). GeSeq - versatile and accurate annotation of organelle genomes. *Nucleic Acids Research*.

[15] Laslett D., Canback B. (2004). ARAGORN, a program to detect tRNA genes and tmRNA genes in nucleotide sequences. *Nucleic Acids Research*.

[16] Greiner S., Lehwark P., Bock R. (2019). OrganellarGenomeDRAW (OGDRAW) version 1.3.1: expanded toolkit for the graphical visualization of organellar genomes. *Nucleic Acids Research*.

[17] Camacho C., Coulouris G., Avagyan V. (2009). BLAST+: architecture and applications. *BMC Bioinformatics*.

[18] Beier S., Thiel T., Münch T., Scholz U., Mascher M. (2017). MISA-web: a web server for microsatellite prediction. *Bioinformatics*.

[19] Katoh K., Standley D. M. (2013). MAFFT multiple sequence alignment software version 7: improvements in performance and usability. *Molecular Biology and Evolution*.

[20] Ronquist F., Teslenko M., van der Mark P. (2012). MrBayes 3.2: efficient Bayesian phylogenetic inference and model choice across a large model space. *Systematic Biology*.

[21] Stamatakis A. (2014). RAxML version 8: a tool for phylogenetic analysis and post-analysis of large phylogenies. *Bioinformatics*.

[22] Lanfear R., Frandsen P. B., Wright A. M., Senfeld T., Calcott B. (2017). PartitionFinder 2: new methods for selecting partitioned models of evolution for molecular and morphological phylogenetic analyses. *Molecular Biology and Evolution*.

[23] Shimodaira H., Hasegawa M. (2001). CONSEL: for assessing the confidence of phylogenetic tree selection. *Bioinformatics*.

[24] Wang D., Zhang Y., Zhang Z., Zhu J., Yu J. (2010). KaKs_Calculator 2.0: a toolkit incorporating gamma-series methods and sliding window strategies. *Genomics, Proteomics & Bioinformatics*.

[25] Frazer K. A., Pachter L., Poliakov A., Rubin E. M., Dubchak I. (2004). VISTA: computational tools for comparative genomics. *Nucleic Acids Research*.

[26] Tiller N., Bock R. (2014). The translational apparatus of plastids and its role in plant development. *Molecular Plant*.

[27] Jansen R. K., Cai Z., Raubeson L. A. (2007). Analysis of 81 genes from 64 plastid genomes resolves relationships in angiosperms and identifies genome-scale evolutionary patterns. *Proceedings of the National Academy of Sciences of the United States of America*.

[28] Daniell H., Lin C. S., Yu M., Chang W. J. (2016). Chloroplast genomes: diversity, evolution, and applications in genetic engineering. *Genome Biology*.

[29] Millen R. S., Olmstead R. G., Adams K. L. (2001). Many parallel losses of infA from chloroplast DNA during angiosperm evolution with multiple independent transfers to the nucleus. *The Plant Cell*.

[30] Gandhi S. G., Awasthi P., Bedi Y. S. (2010). Analysis of SSR dynamics in chloroplast genomes of Brassicaceae family. *Bioinformation*.

[31] Song S.-L., Lim P.-E., Phang S.-M., Lee W.-W., Hong D., Prathep A. (2014). Development of chloroplast simple sequence repeats (cpSSRs) for the intraspecific study of Gracilaria tenuistipitata (Gracilariales, Rhodophyta) from different populations. *BMC Research Notes*.

[32] Diekmann K., Hodkinson T. R., Barth S. (2012). New chloroplast microsatellite markers suitable for assessing genetic diversity of Lolium perenne and other related grass species. *Annals of Botany*.

[33] Wheeler G. L., Dorman H. E., Buchanan A., Challagundla L., Wallace L. E. (2014). A review of the prevalence, utility, and caveats of using chloroplast simple sequence repeats for studies of plant biology. *Applications in Plant Sciences*.

[34] Berger B. A., Kriebel R., Spalink D., Sytsma K. J. (2016). Divergence times, historical biogeography, and shifts in speciation rates of Myrtales. *Molecular Phylogenetics and Evolution*.

[35] Lin N., Moore M. J., Deng T. (2018). Complete plastome sequencing from Toona (Meliaceae) and phylogenomic analyses within Sapindales. *Applications in Plant Sciences*.

[36] Wang Y., Zhan D.-F., Jia X. (2016). Complete chloroplast genome sequence of Aquilaria sinensis (Lour.) Gilg and evolution analysis within the Malvales order. *Frontiers in Plant Science*.

[37] Zhao K. K., Wang J. H., Cai Y.-C., Zhu Z.-X., López-Pujol J., Wang H.-F. (2018). Complete chloroplast genome sequence of Heritiera angustata(Malvaceae): an endangered plant species. *Mitochondrial DNA Part B*.

[38] Xi Z., Ruhfel B. R., Schaefer H. (2012). Phylogenomics and a posteriori data partitioning resolve the Cretaceous angiosperm radiation Malpighiales. *Proceedings of the National Academy of Sciences of the United States of America*.

[39] de Santana Lopes A., Pacheco T. G., Santos K. G. D. (2018). The Linum usitatissimum L. plastome reveals atypical structural evolution, new editing sites, and the phylogenetic position of Linaceae within Malpighiales. *Plant Cell Reports*.

[40] Du Y.-p., Bi Y., Yang F.-p. (2017). Complete chloroplast genome sequences of Lilium : insights into evolutionary dynamics and phylogenetic analyses. *Scientific Reports*.

[41] Wang X., Zhou T., Bai G., Zhao Y. (2018). Complete chloroplast genome sequence of Fagopyrum dibotrys: genome features, comparative analysis and phylogenetic relationships. *Scientific Reports*.

[42] Jeon J. H., Kim S.-C. (2019). Comparative analysis of the complete chloroplast genome sequences of three closely related East-Asian wild roses (Rosa sect. Synstylae; Rosaceae). *Genes*.

[43] Kimura M. (1989). The neutral theory of molecular evolution and the world view of the neutralists. *Genome*.

[44] Menezes A. P. A., Resende-Moreira L. C., Buzatti R. S. O. (2018). Chloroplast genomes of Byrsonima species (Malpighiaceae): comparative analysis and screening of high divergence sequences. *Scientific Reports*.

[45] Schöttler M. A., Thiele W., Belkius K. (2017). The plastid-encoded PsaI subunit stabilizes photosystem I during leaf senescence in tobacco. *Journal of Experimental Botany*.

[46] Fleischmann T. T., Scharff L. B., Alkatib S., Hasdorf S., Schöttler M. A., Bock R. (2011). Nonessential plastid-encoded ribosomal proteins in tobacco: a developmental role for plastid translation and implications for reductive genome evolution. *Plant Cell*.

[47] Ivanova Z., Sablok G., Daskalova E. (2017). Chloroplast genome analysis of resurrection tertiary relict Haberlea rhodopensis highlights genes important for desiccation stress response. *Frontiers in Plant Science*.

[48] Wang W., Yu H., Wang J. (2017). The complete chloroplast genome sequences of the medicinal plant Forsythia suspensa (Oleaceae). *International Journal of Molecular Sciences*.

[49] Shi C., Han K., Li L. (2019). Complete chloroplast genomes of 14 mangroves: phylogenetic and genomic comparative analyses. *bioRxiv*.

