# Supplementary material for: Complete Chloroplast Genomes of 14 Mangroves: Phylogenetic and Comparative Genomic Analyses
Source: Biomed Res Int. 2020 May 5;2020:8731857. doi: 10.1155/2020/8731857 (PMC7225854; doi:10.1155/2020/8731857)
Supplement: Supplementary Materials — Figure S1: the whole chloroplast genomes of 14 mangroves. The inner circle marks the LSC, SSC, and IR regions. Genes' position and orientations are shown along the outer circle. Genes with different functions are colored. Figure S2: the SSR distribution in the 14 mangroves and 57 terrestrial plant chloroplast genomes. (A) Number of different SSR types in each species. Mangroves are marked in red. (B) The SSR numbers in 17 orders (dots for each species and bars for average number). Figure S3: the ML phylogenetic tree based on whole chloroplast genes of 14 mangroves and 57 land species. Figure S4: the BI phylogenetic tree based on whole chloroplast genes of 14 mangroves and 57 land species using partition model. Figure S5: the ML phylogenetic tree based on whole chloroplast genes of 14 mangroves and 57 land species using partition model. Figure S6: the BI phylogenetic tree based on four conserved genes (ndhF, matK, rbcL, and atpB) of 14 mangroves and 57 land species. Figure S7: the ML phylogenetic tree based on four conserved genes (ndhF, matK, rbcL, and atpB) of 14 mangroves and 57 land species. Figure S8: the genomic comparison and similarities of whole chloroplast sequences among mangroves and their related species within orders Lamiales, Fabales, Malpighiales, Malvales, Myrtales, and Sapindales. CNS: conserved noncoding sequence. Figure S9: the Ka/Ks values of chloroplast protein-coding genes in species from Oxalidales, Celastrales, Fagales, Cucurbitales, and Rosales (Rosid I clades). Figure S10: the Ka/Ks values of chloroplast protein-coding genes in species from Brassicales (Rosid II), Huerteales (Rosid II), Geraniales, and Saxifragales. Table S1: GC content in the 14 mangrove chloroplast genomes. Table S2: the 57 public terrestrial species used for genomic comparison analyses (SSR, phylogeny, and evolution). Table S3: the assessment and comparison of phylogenetic trees. [file 8731857.f1.docx]

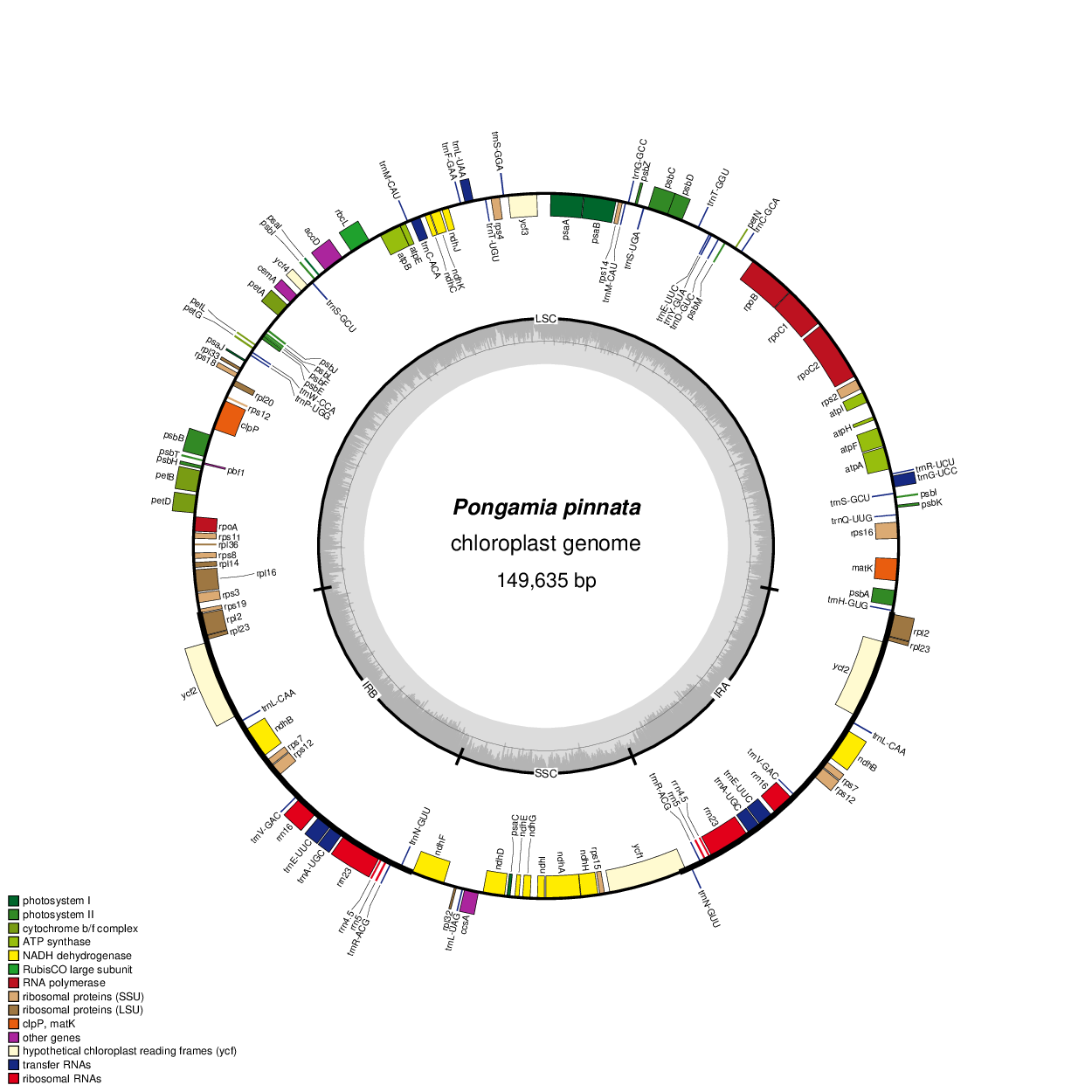


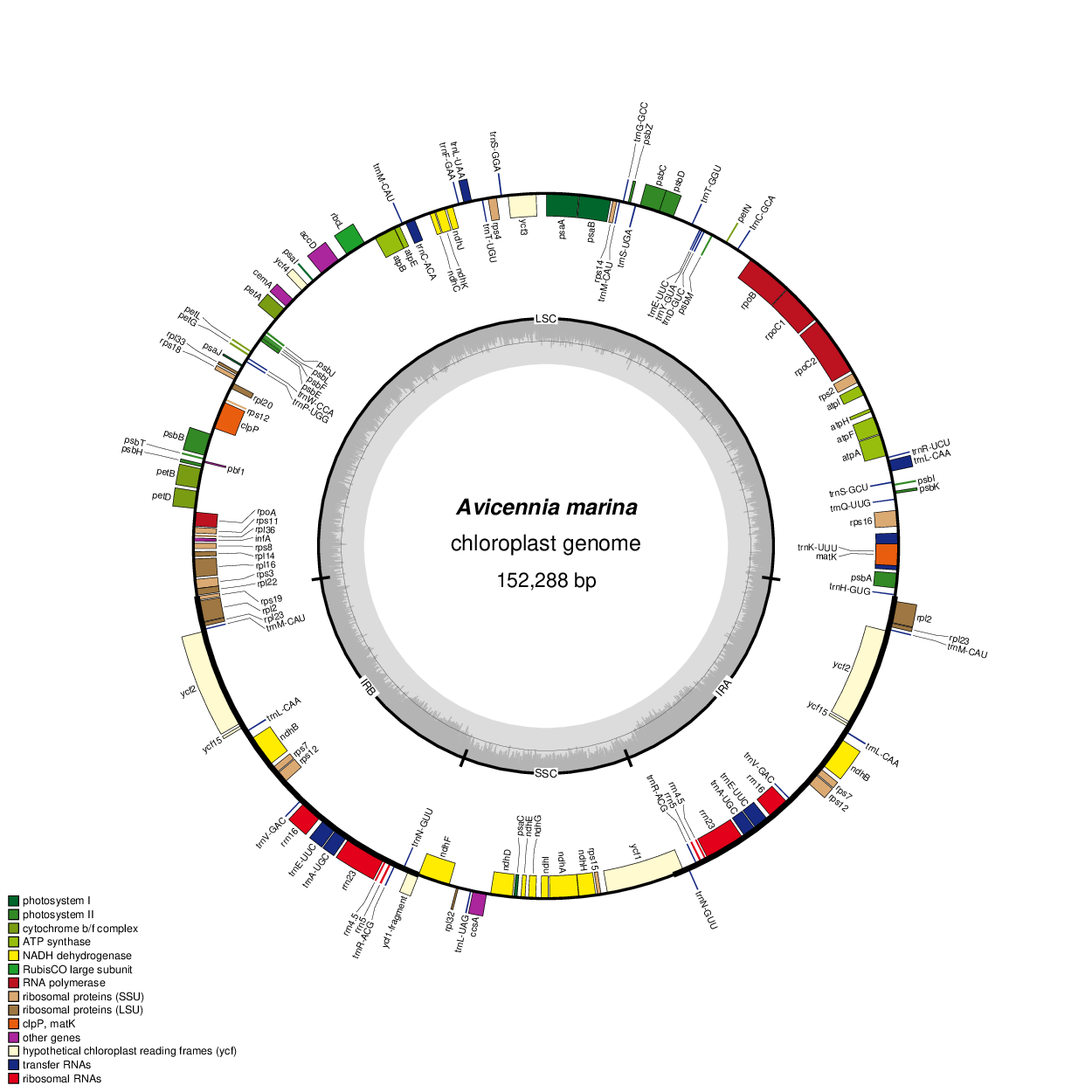


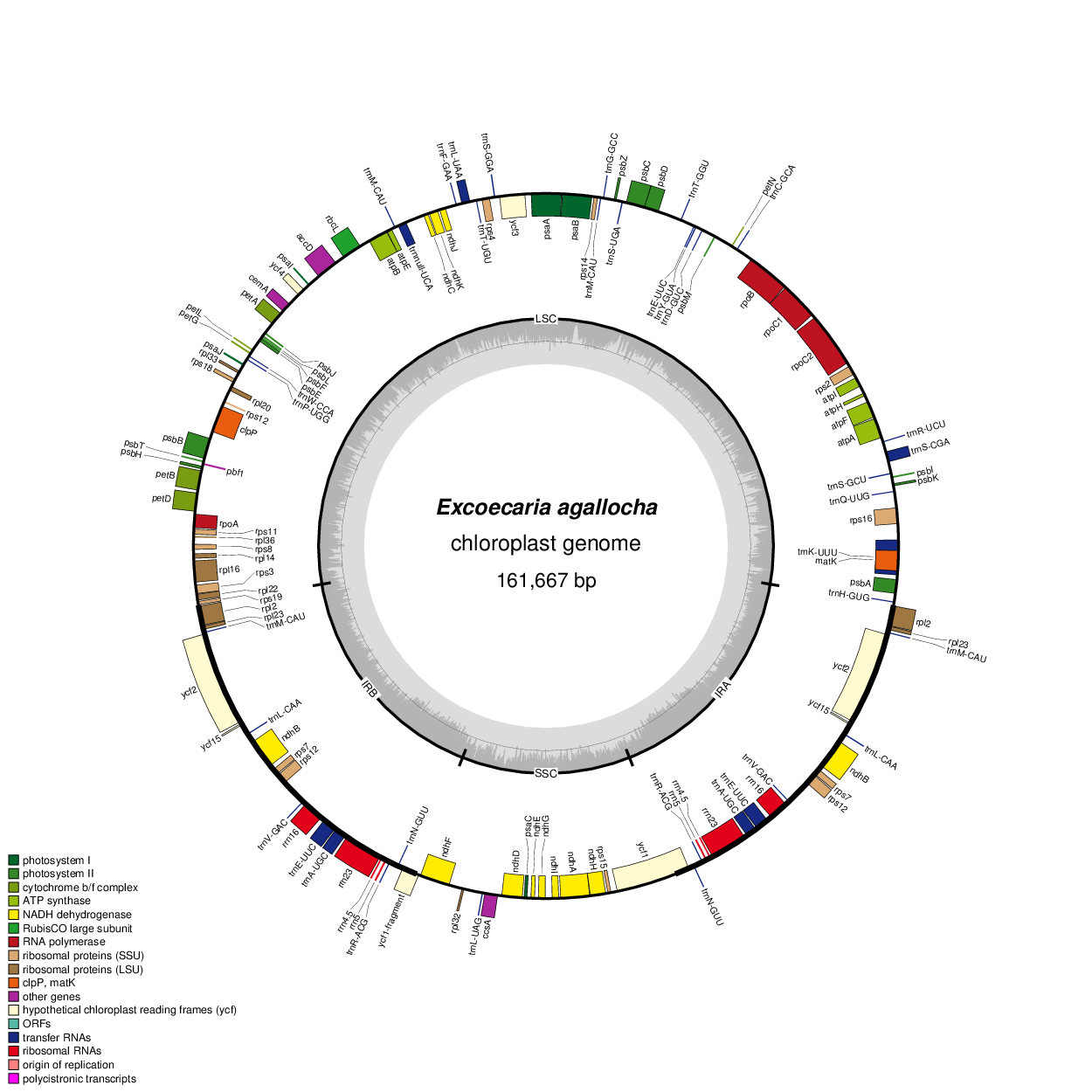


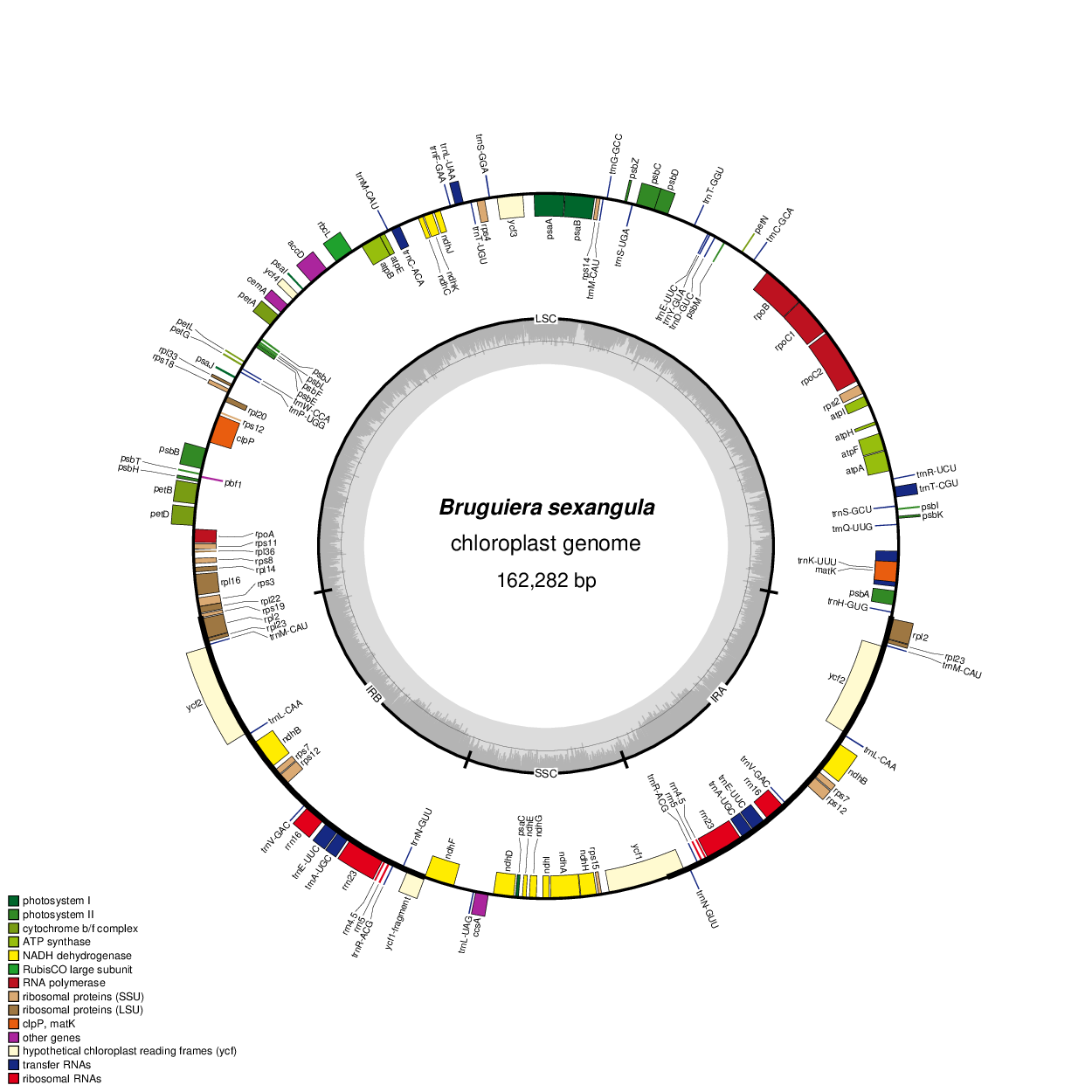


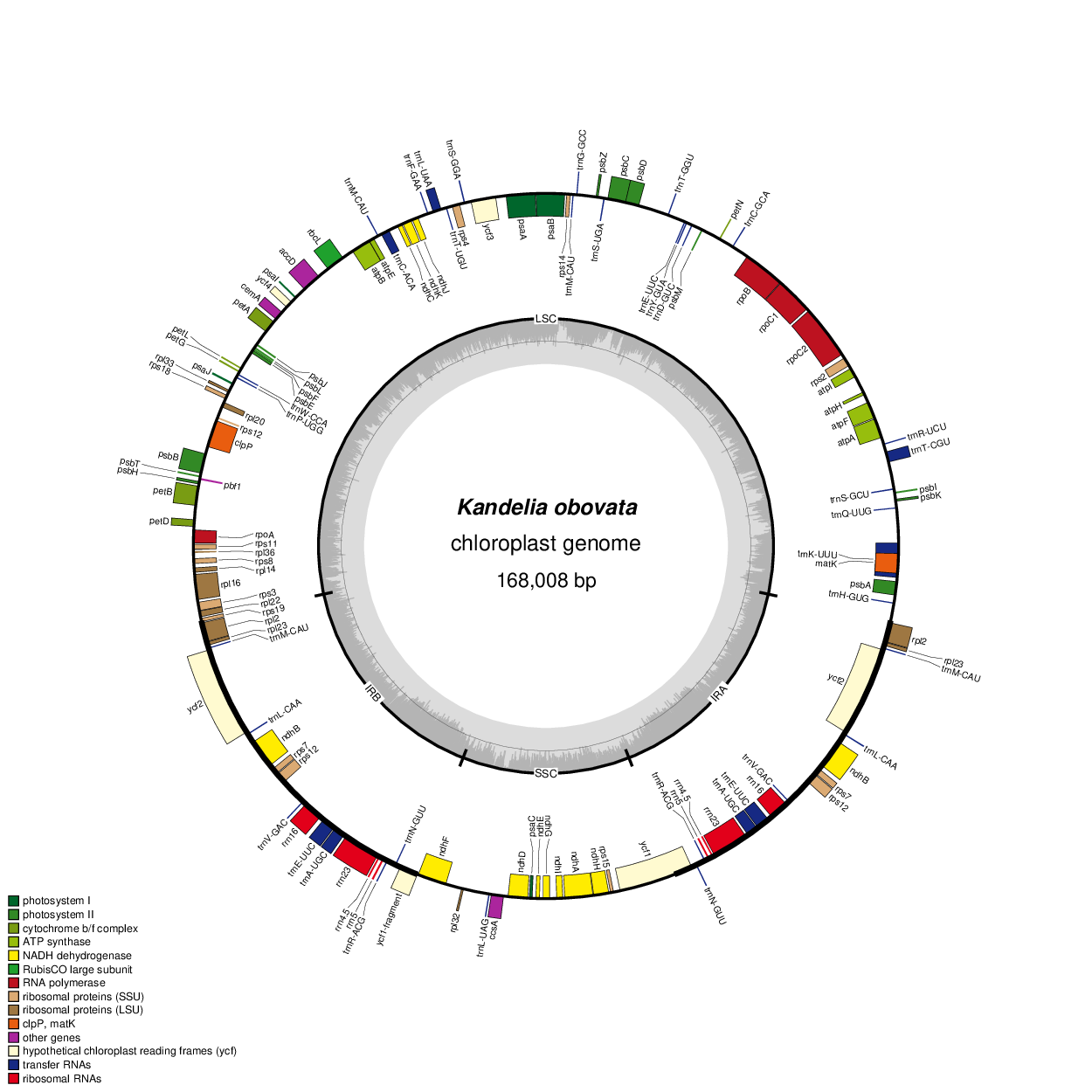


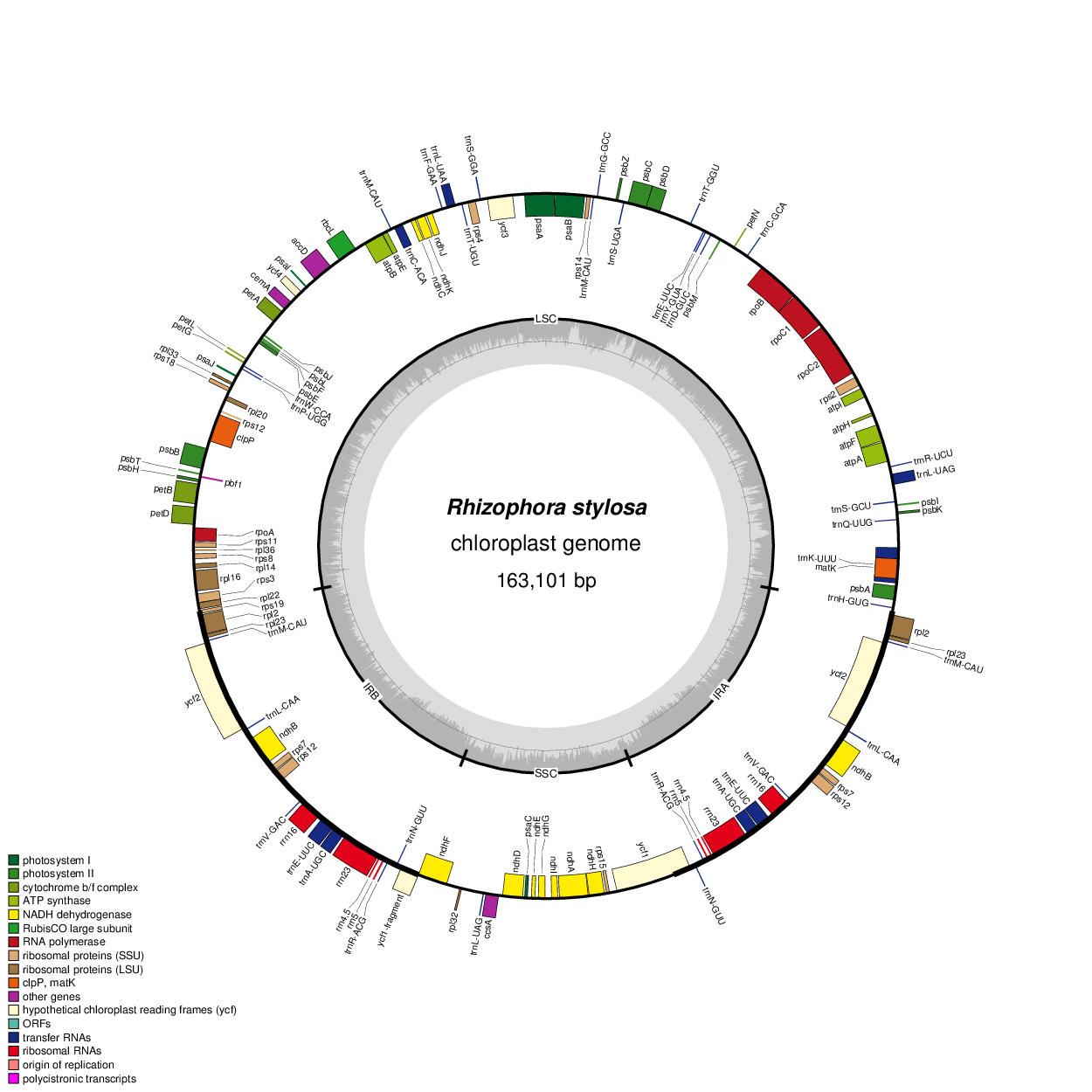


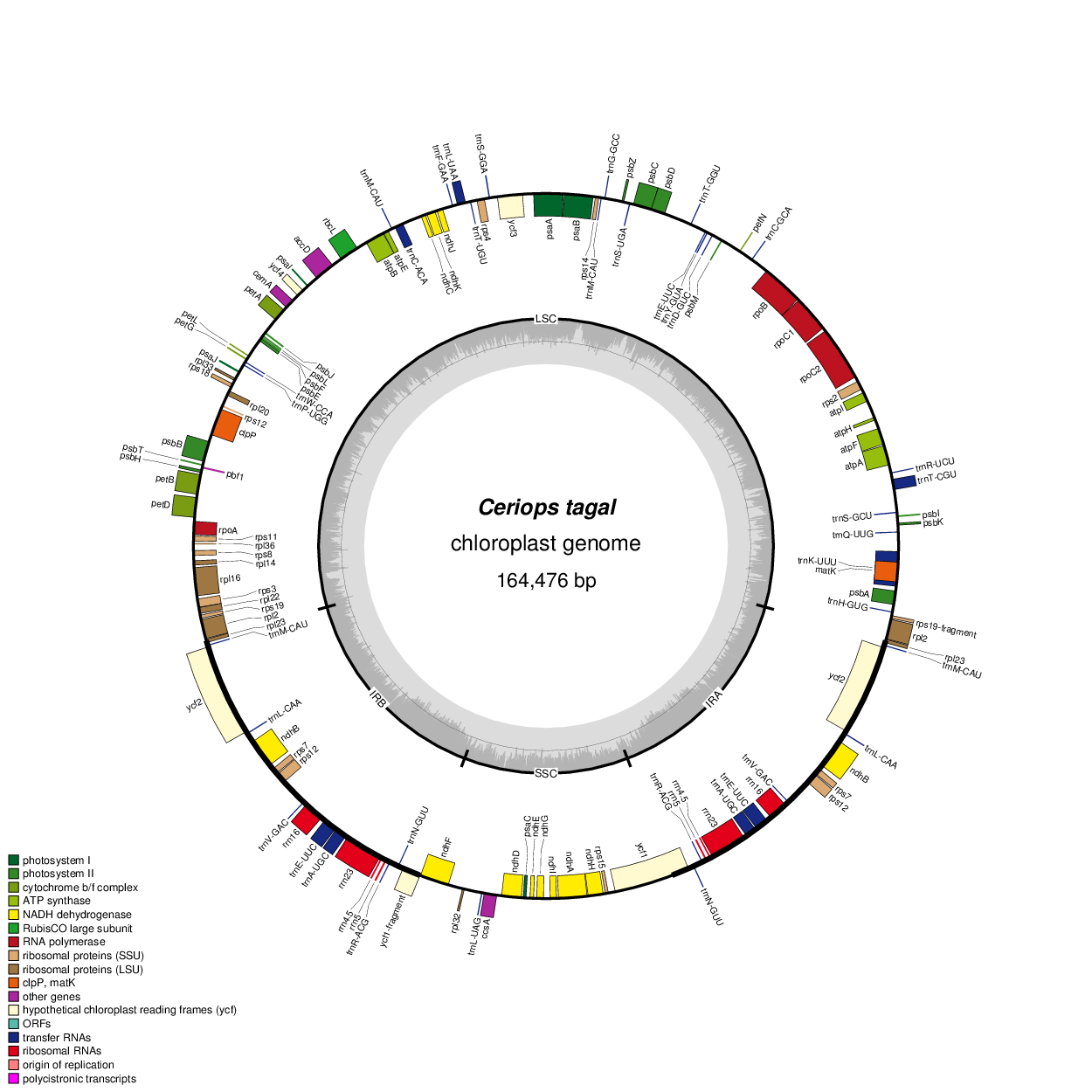


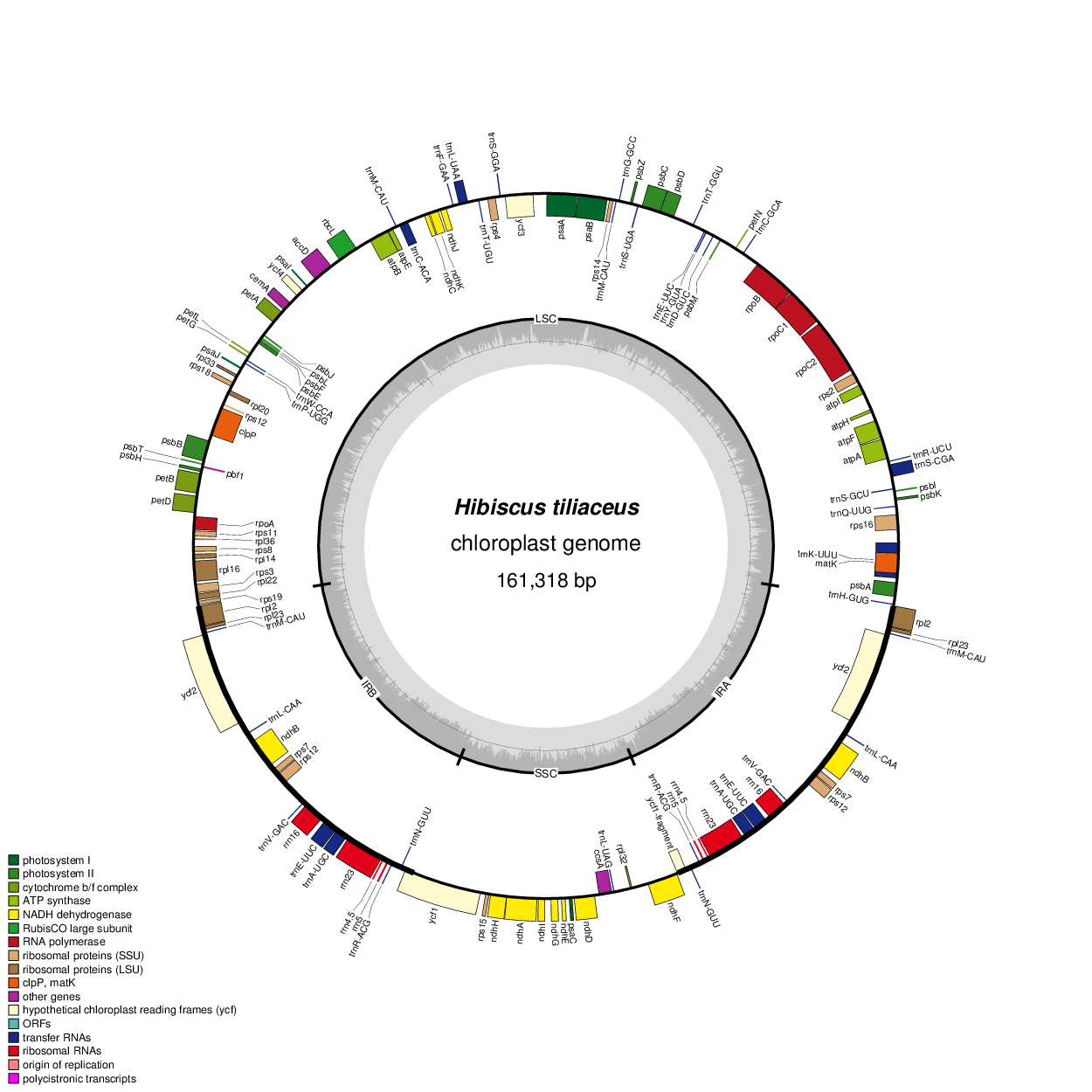


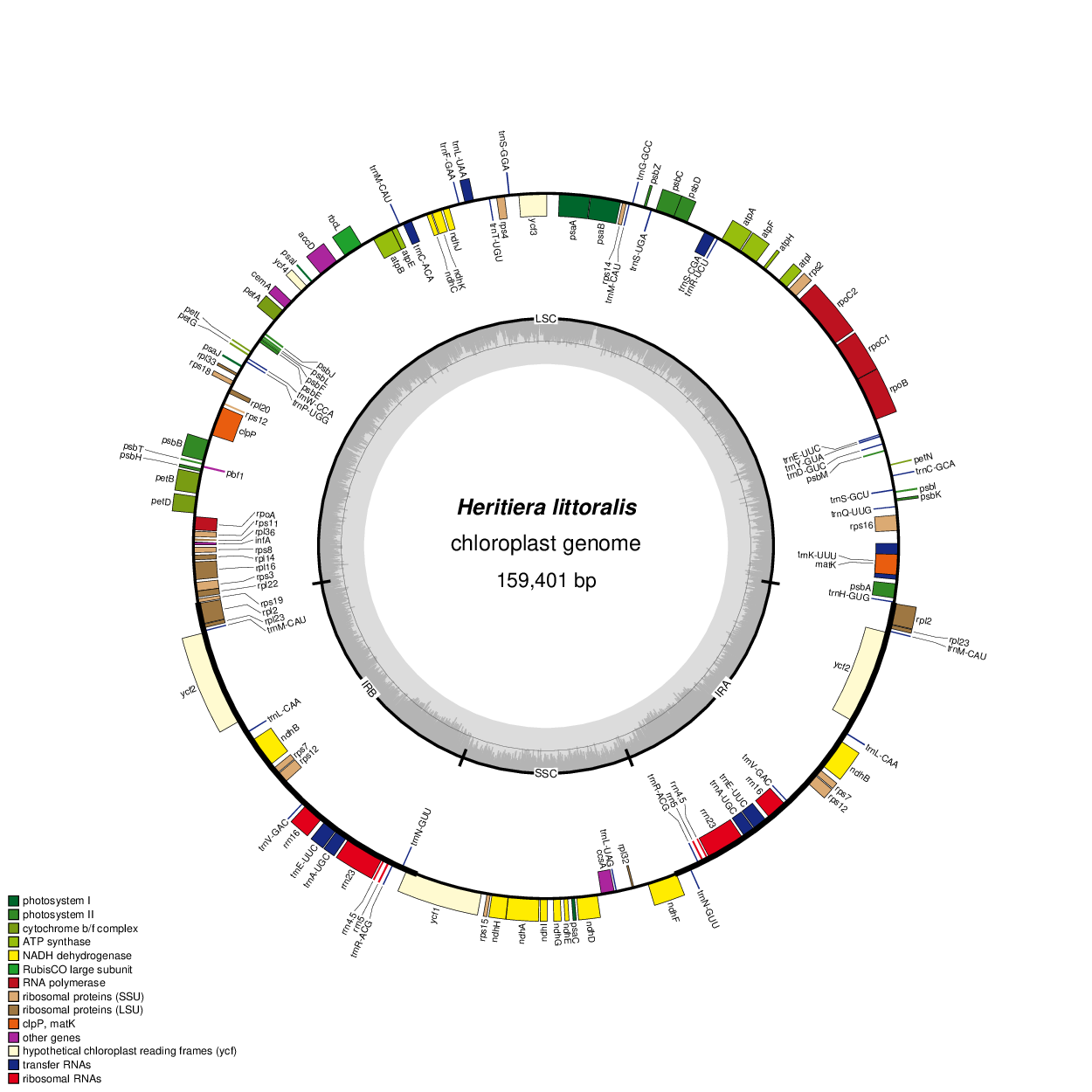


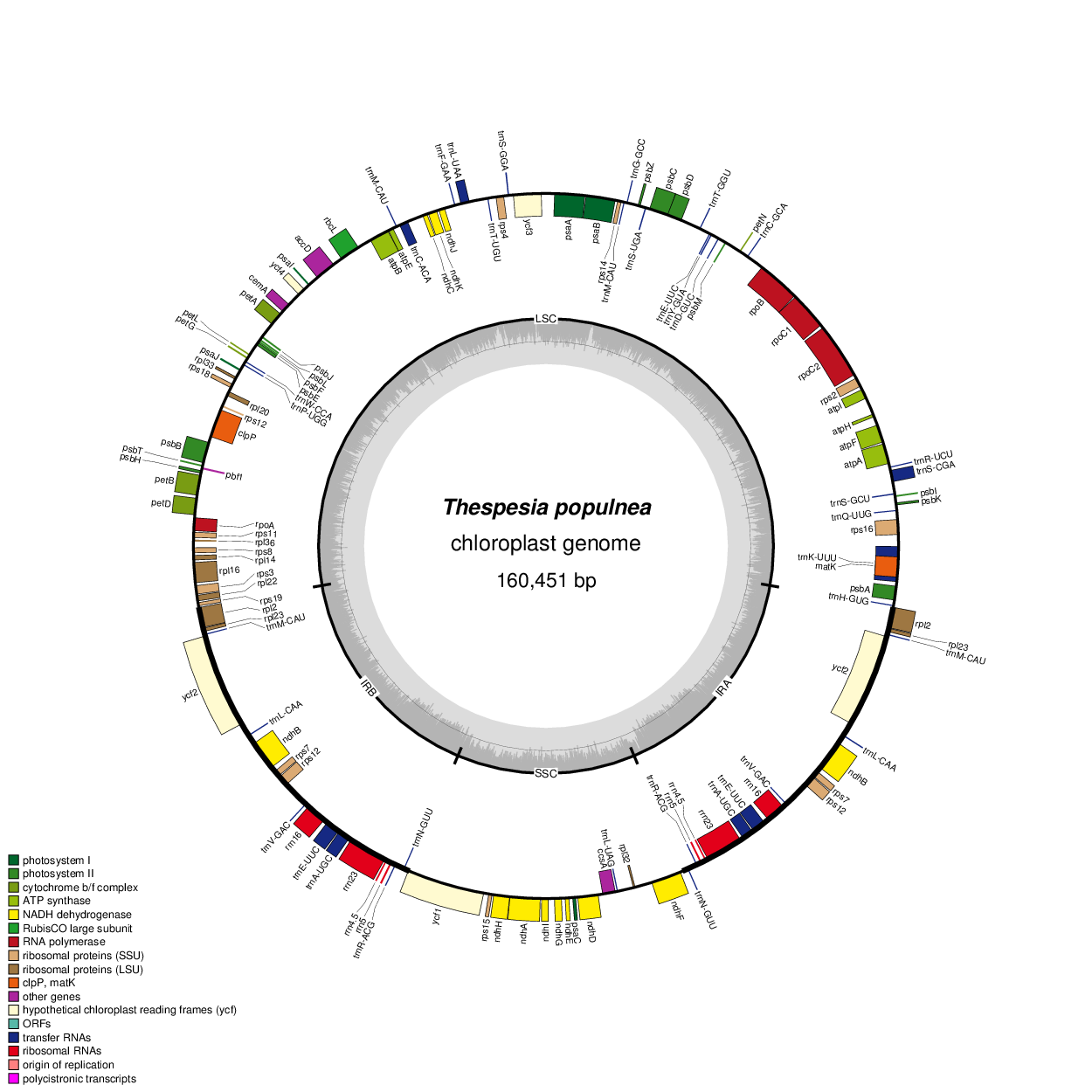


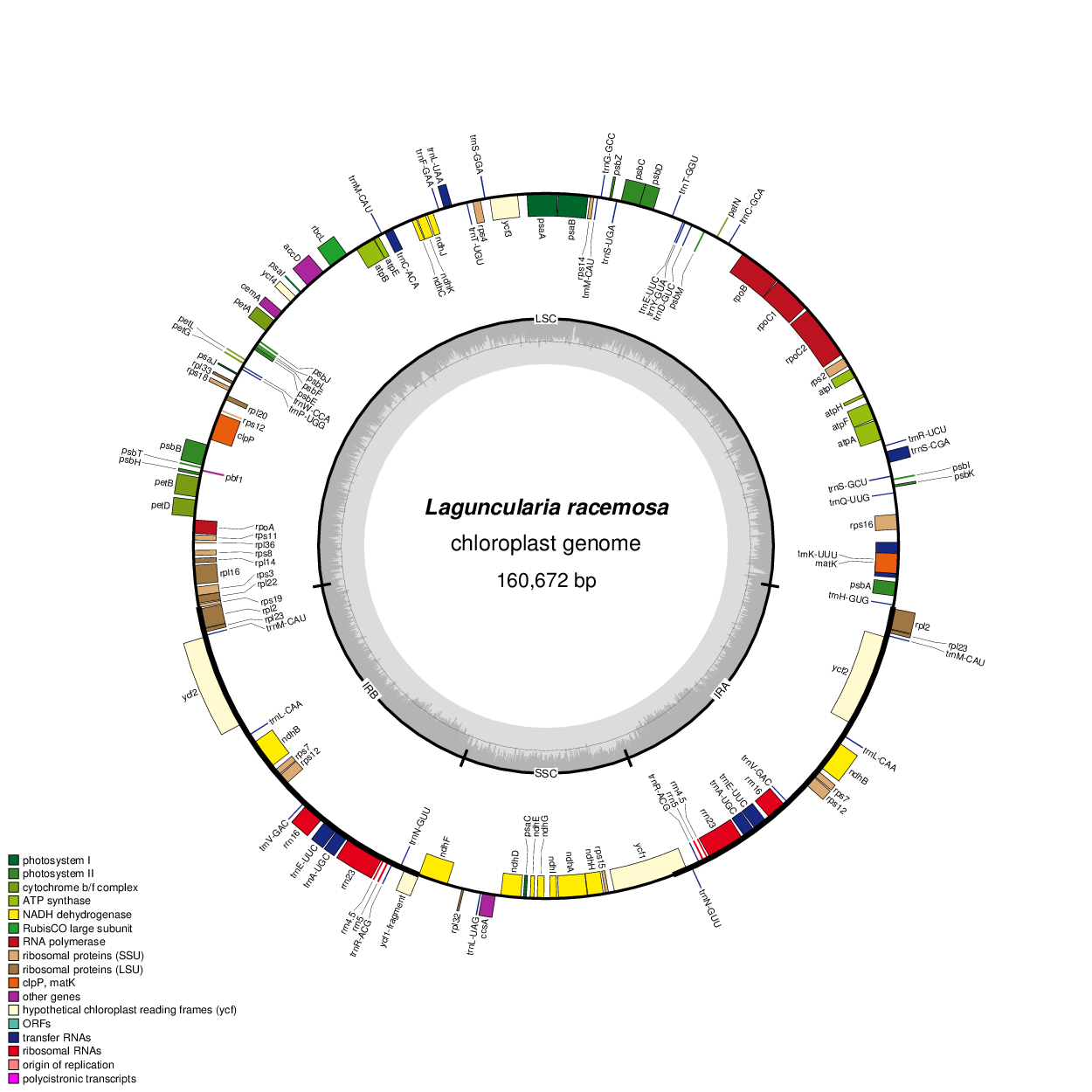


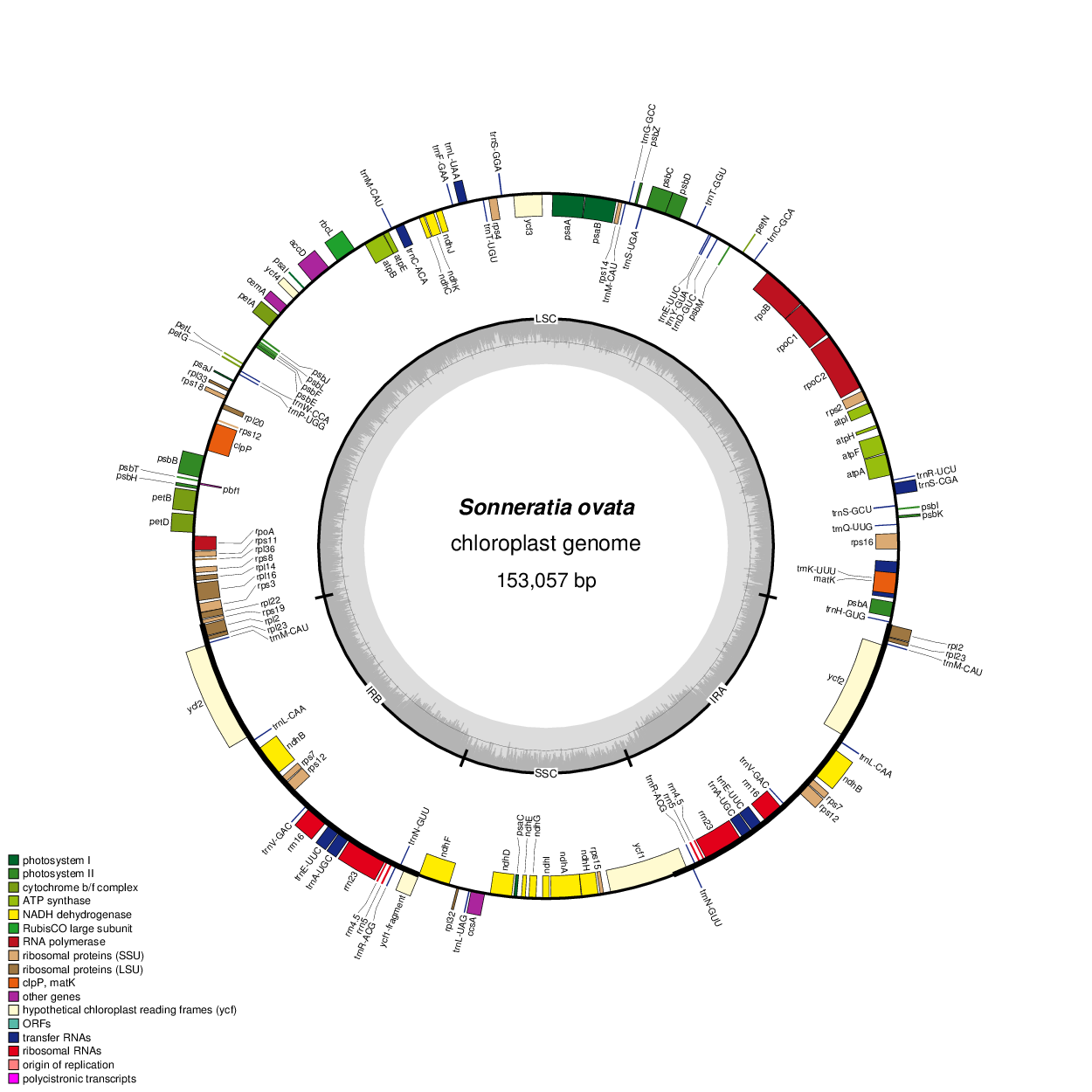


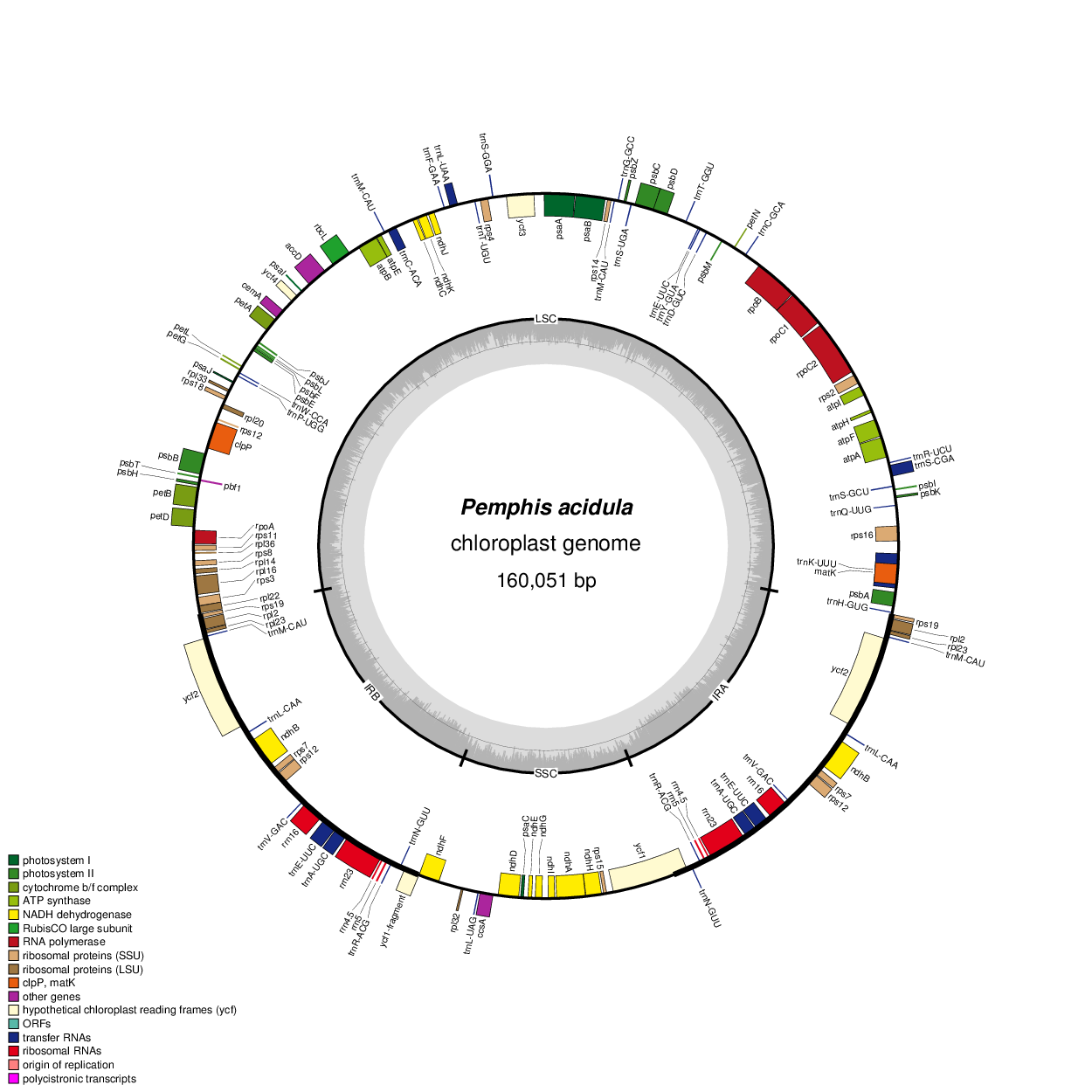


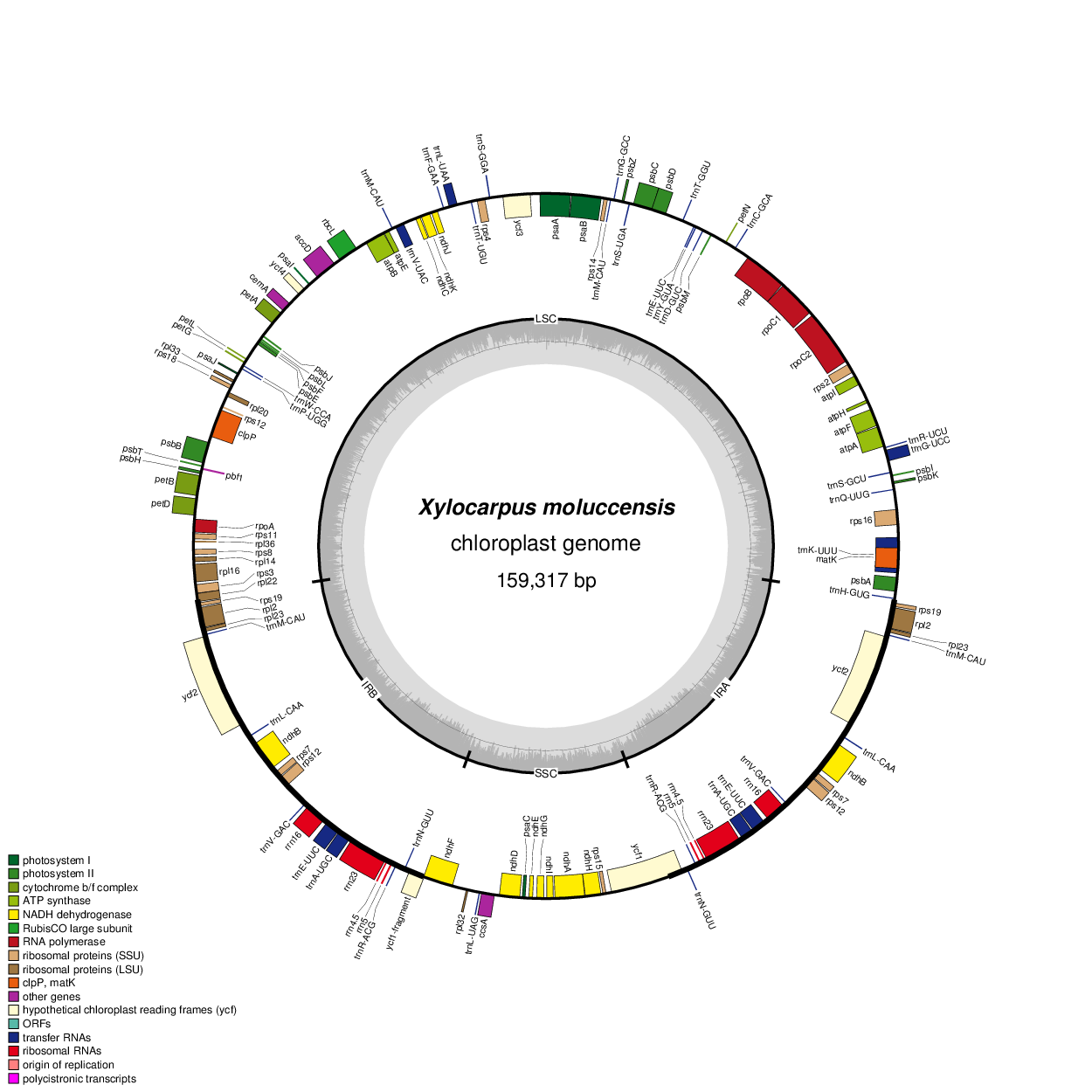


**Figure S1.** The whole chloroplast genomes of 14 mangroves. The inner circle marks the LSC, SSC and IR regions. Genes’ position and orientations are showed along the outer circle. Genes with different functions are colored.


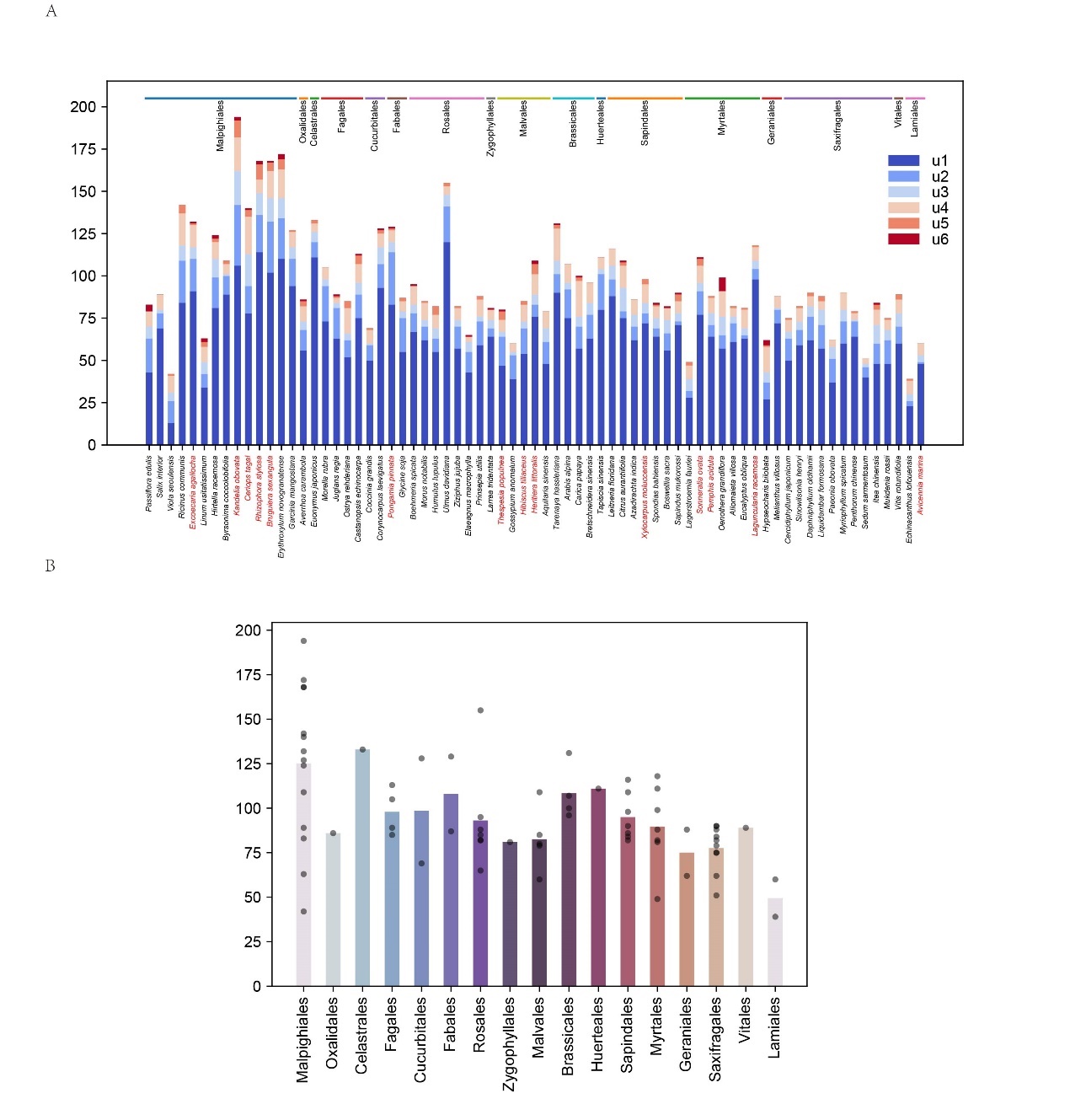


**Figure S2**. The SSR distribution in the 14 mangroves and 57 terrestrial plant chloroplast genomes. (A) Number of different SSR types in each species. Mangroves are marked in red. (B) The SSR numbers in 17 orders (dots for each species and bars for average number).


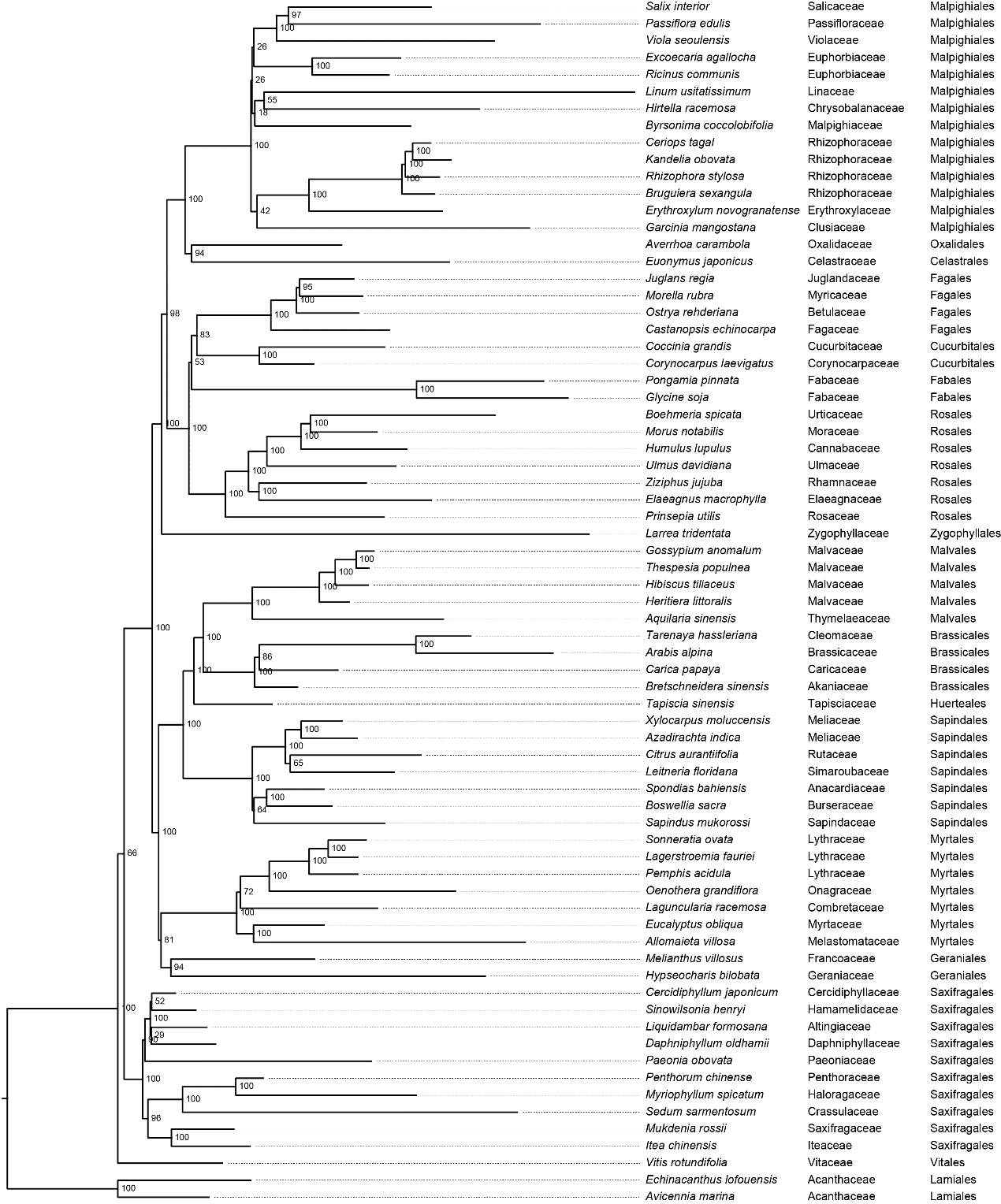


**Figure S3.** The ML phylogenetic tree based on whole chloroplast genes of 14 mangroves and 57 land species.


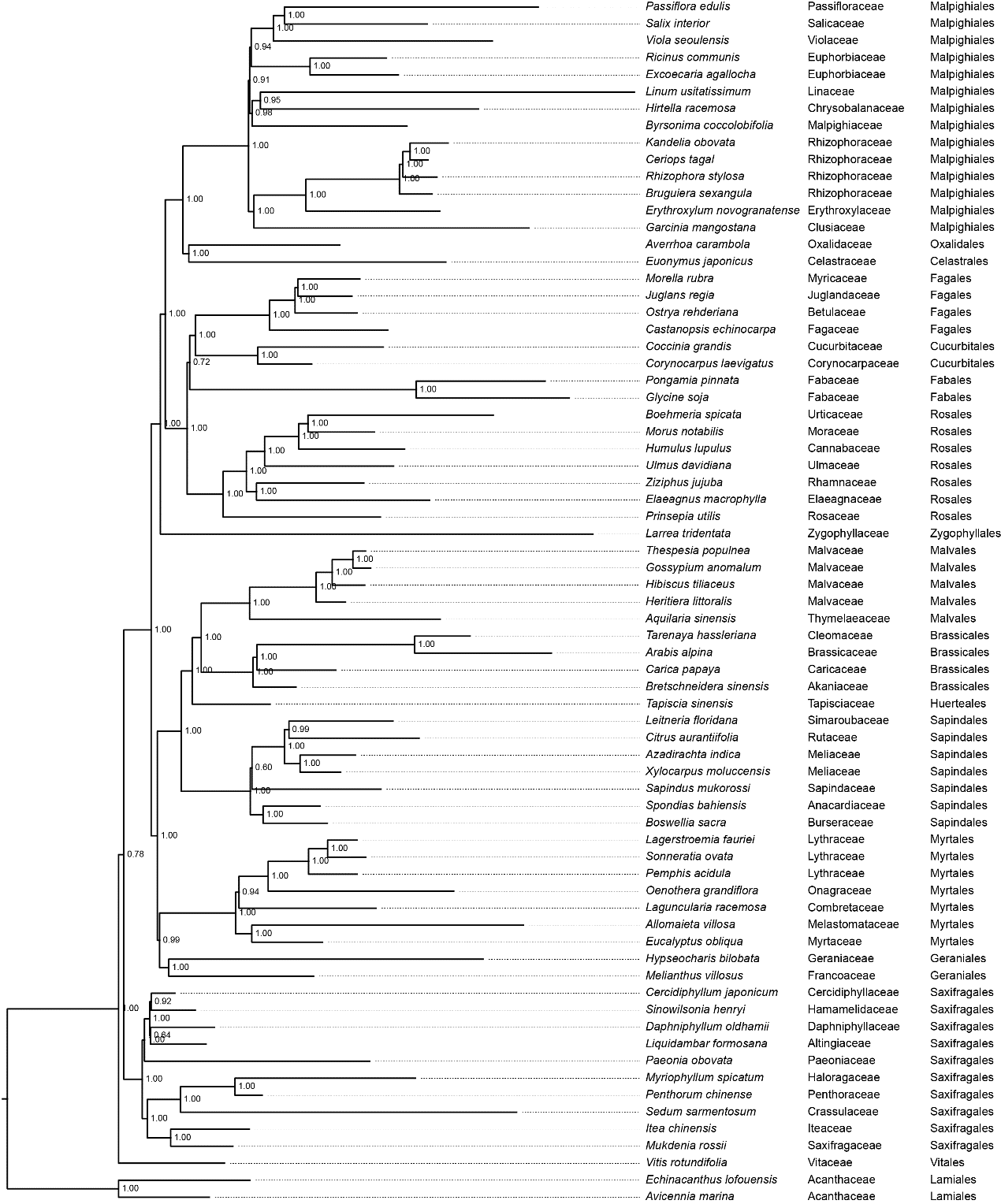


**Figure S4.** The BI phylogenetic tree based on whole chloroplast genes of 14 mangroves and 57 land species using partition model.


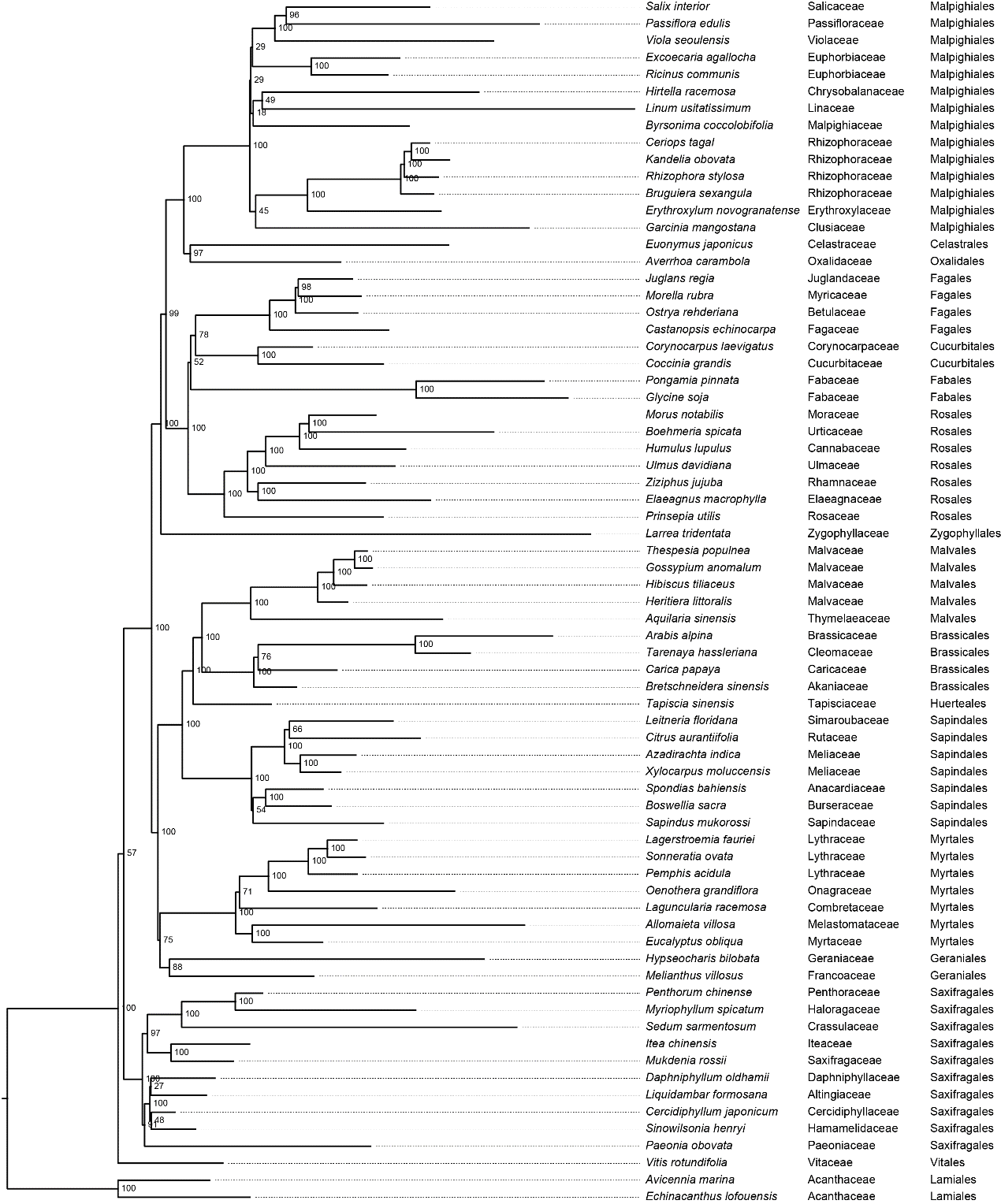


**Figure S5.** The ML phylogenetic tree based on whole chloroplast genes of 14 mangroves and 57 land species using partition model.

**
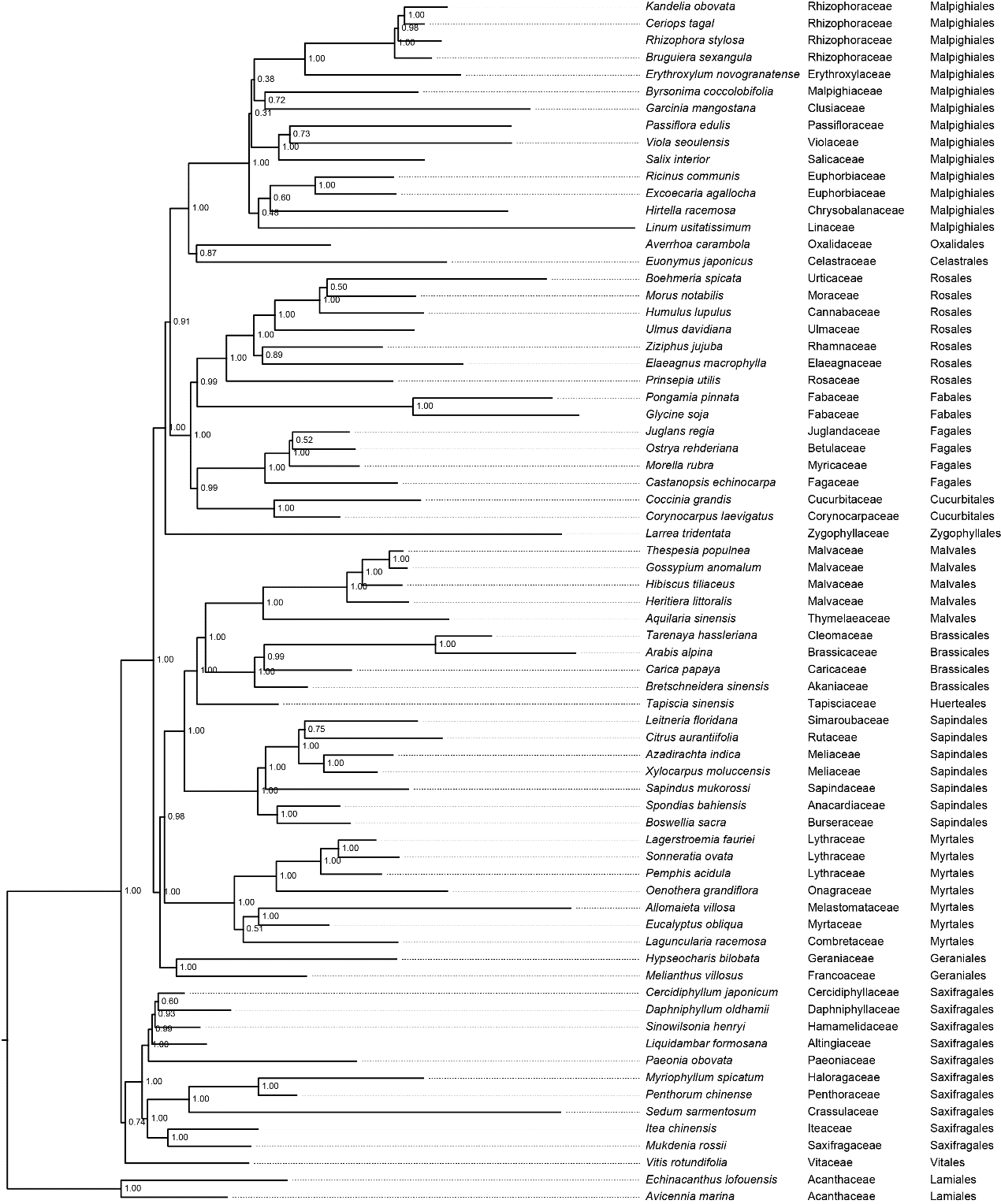
**

**Figure S6.** The BI phylogenetic tree based on four conserved genes (*ndhF*, *matK*, *rbcL*, *atpB*) of 14 mangroves and 57 land species.


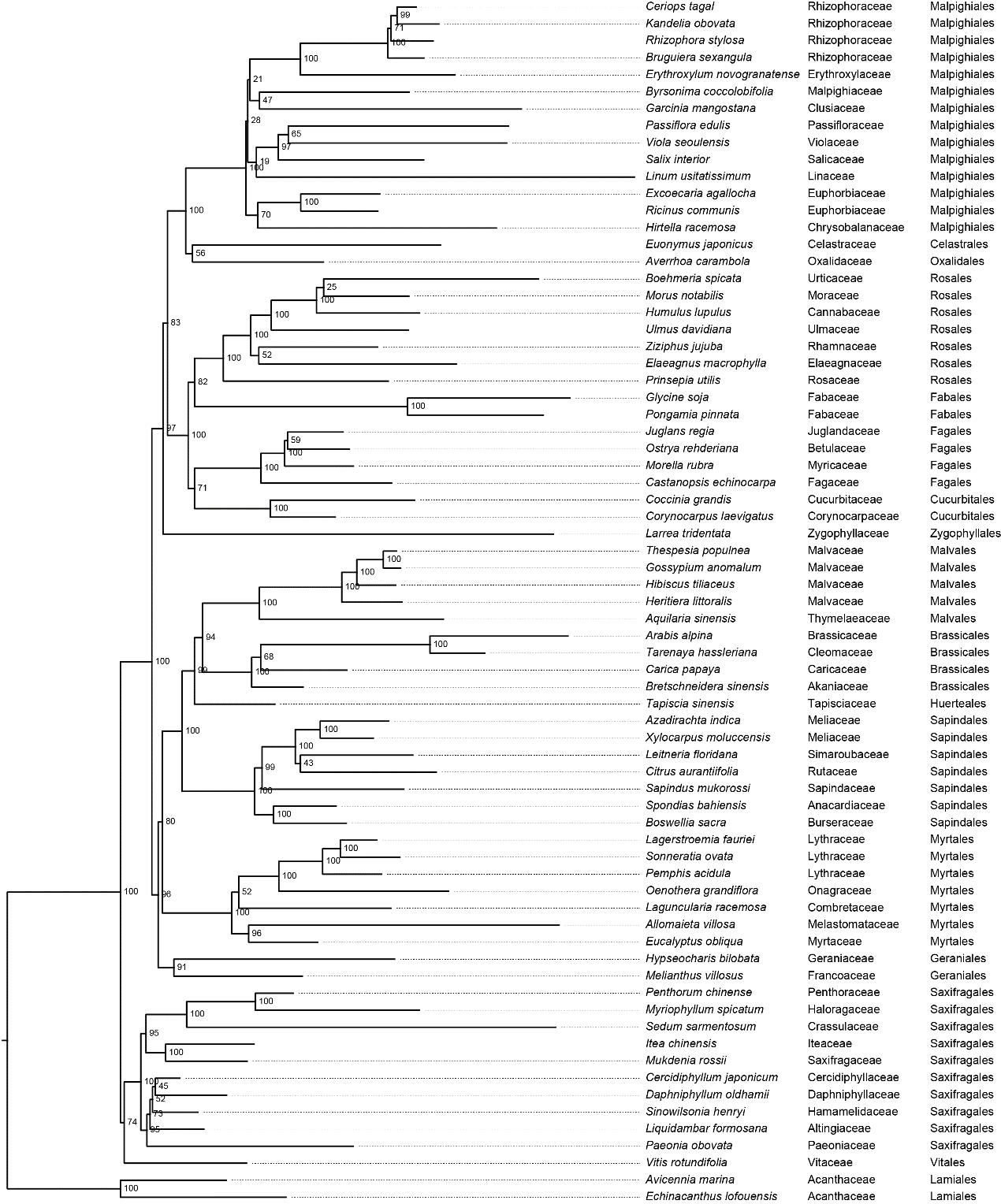


**Figure S7.** The ML phylogenetic tree based on four conserved genes (*ndhF*, *matK*, *rbcL*, *atpB*) of 14 mangroves and 57 land species.


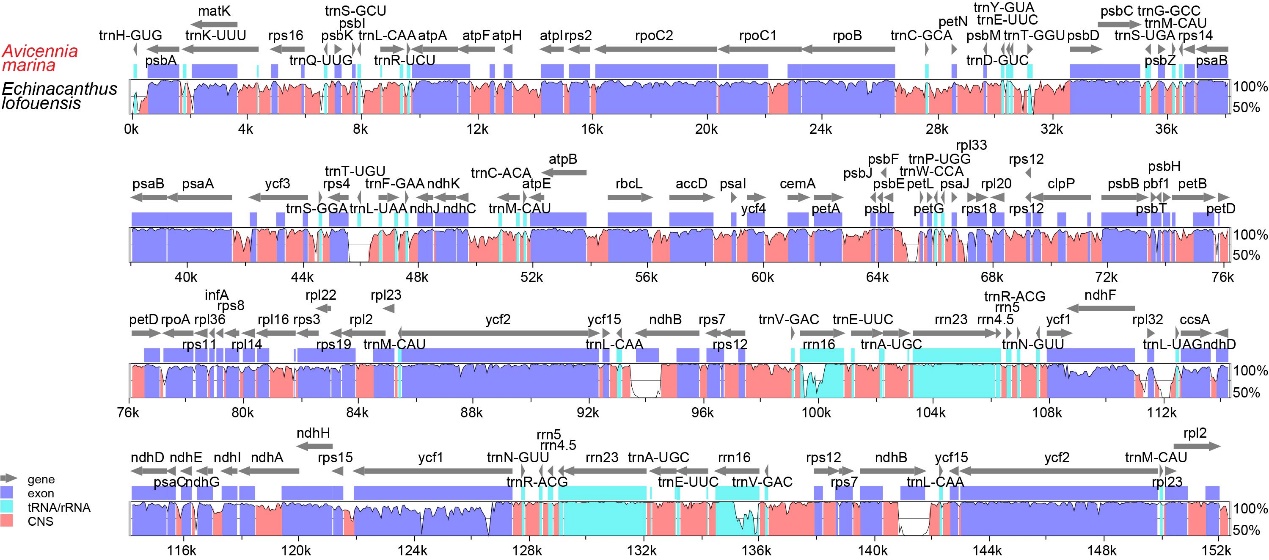


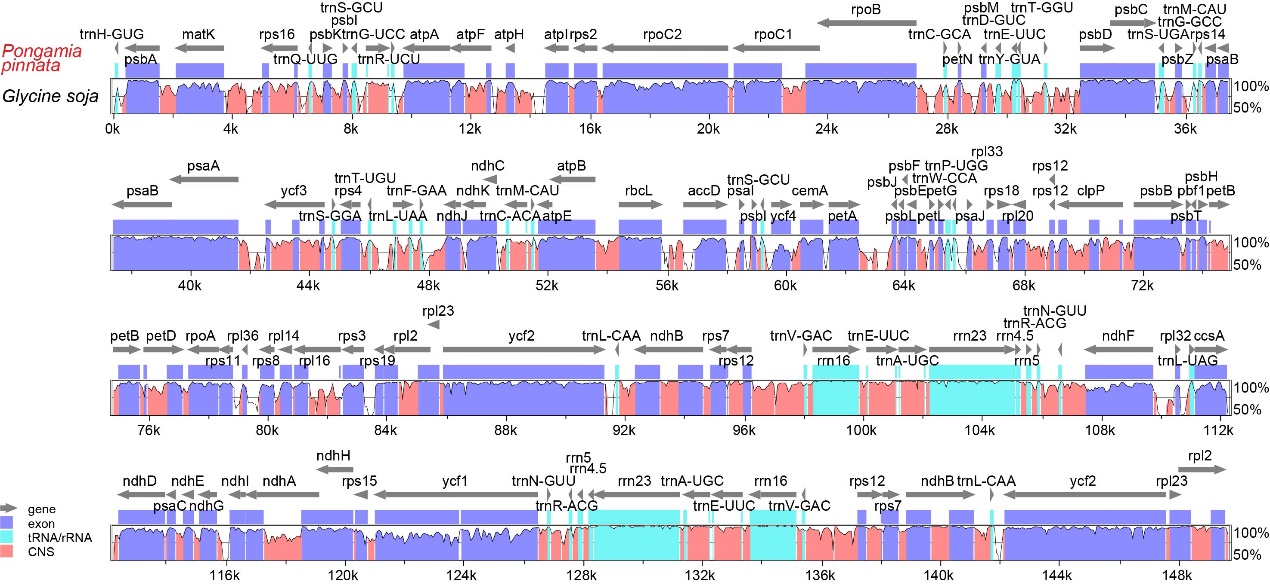


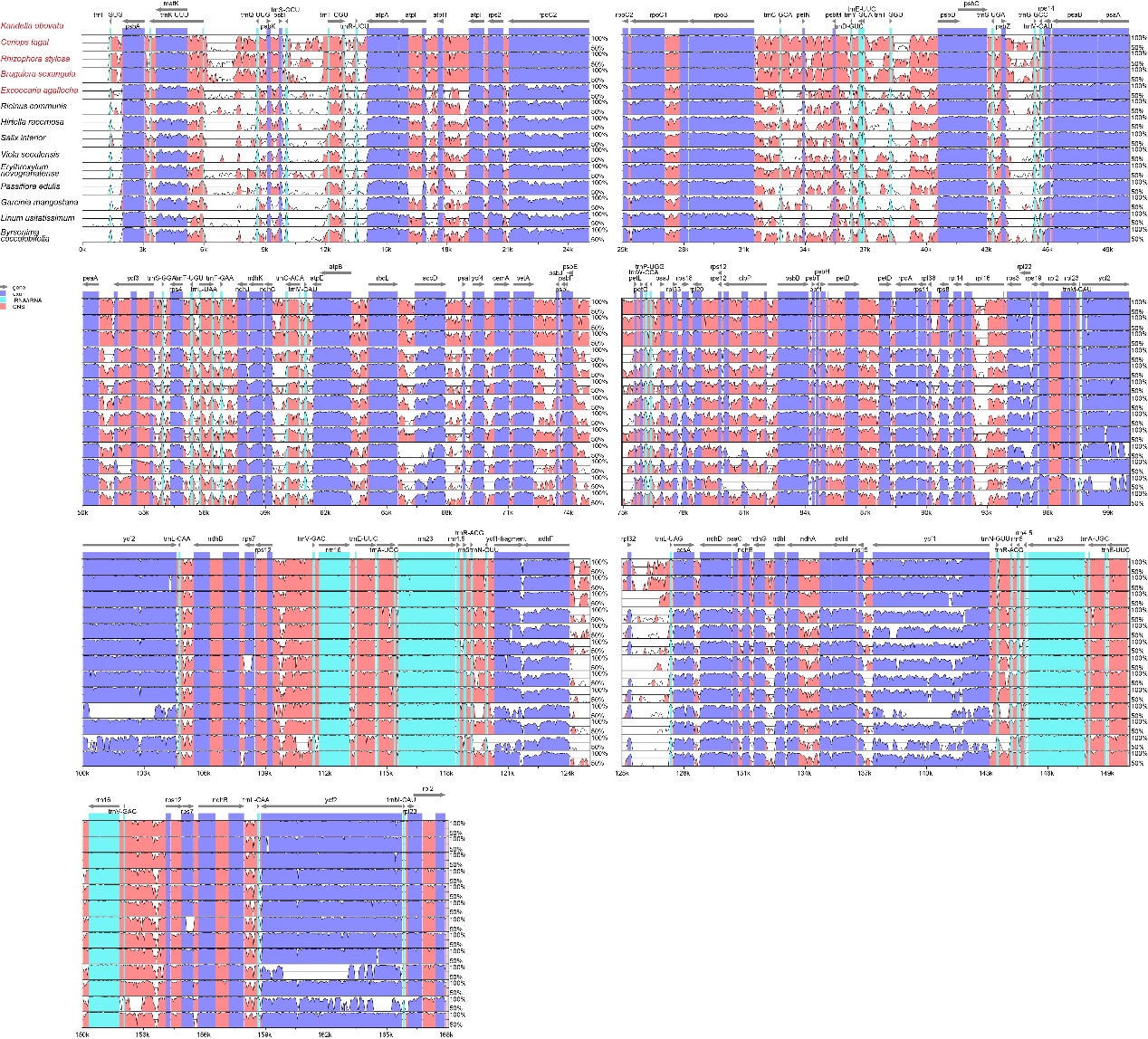


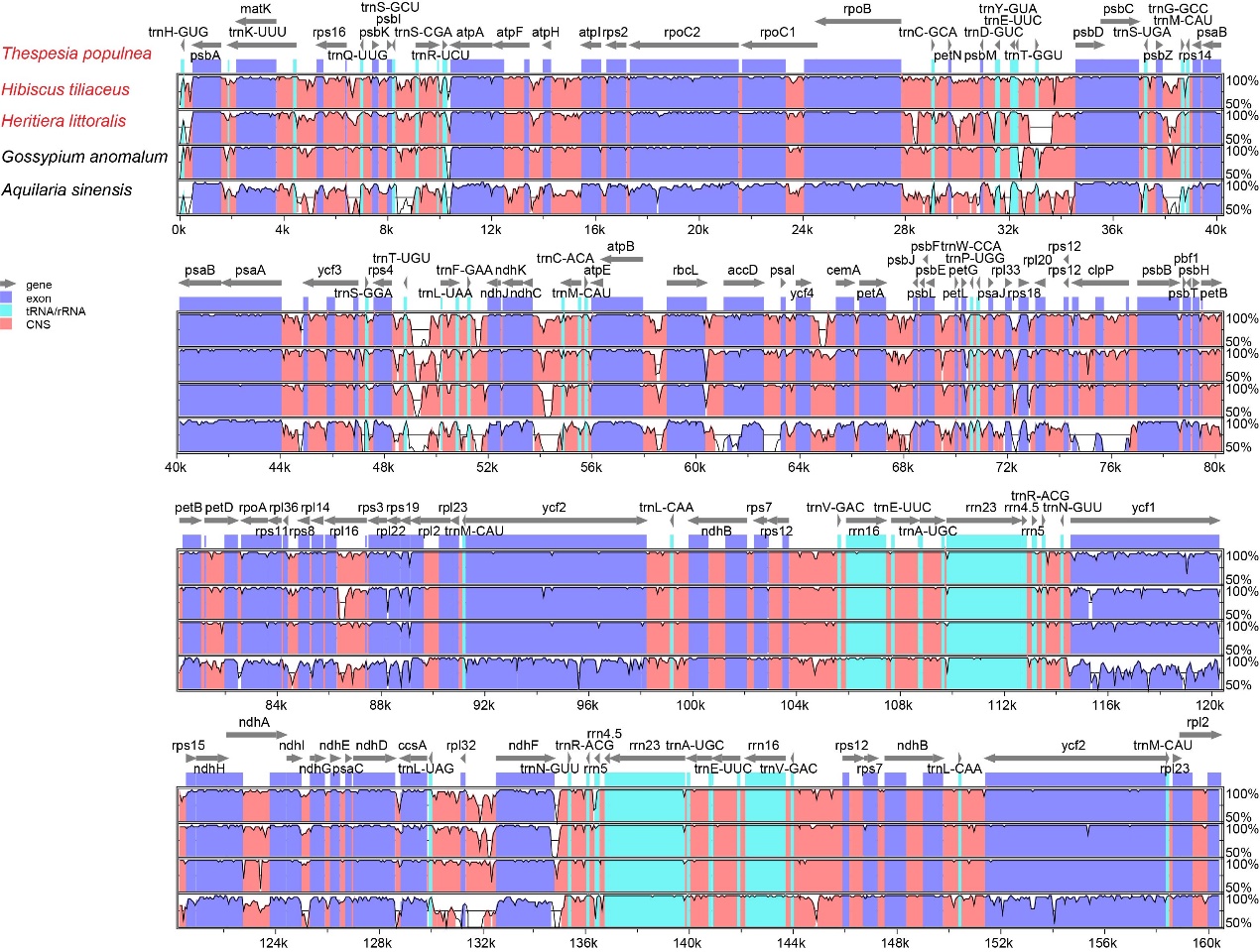


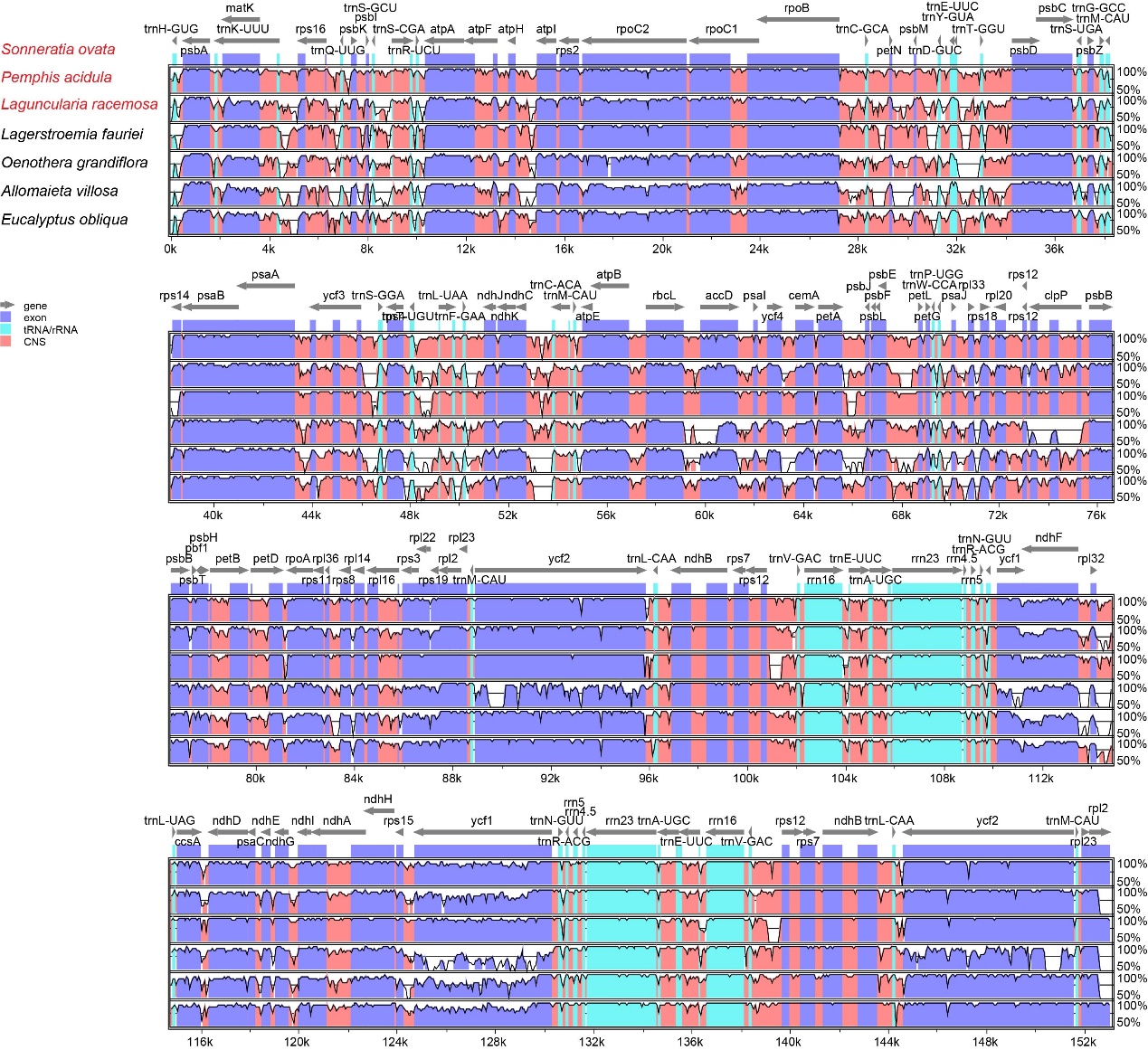


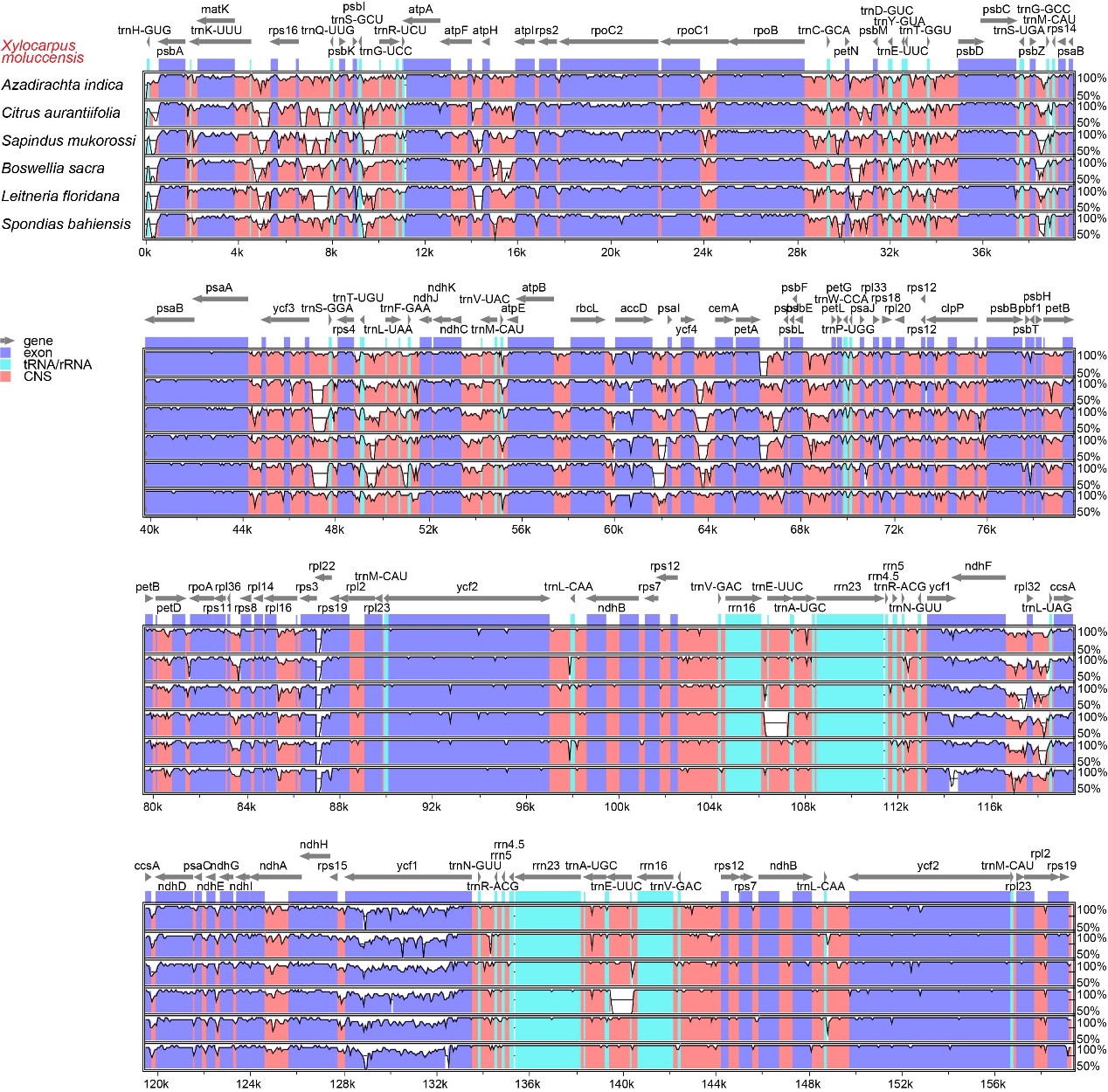


**Figure S8**. The genomic comparison and similarities of whole chloroplast sequences among mangroves and their related species within orders Lamiales, Fabales, Malpighiales, Malvales, Myrtales, and Sapindales. CNS: conserved non-coding sequence.


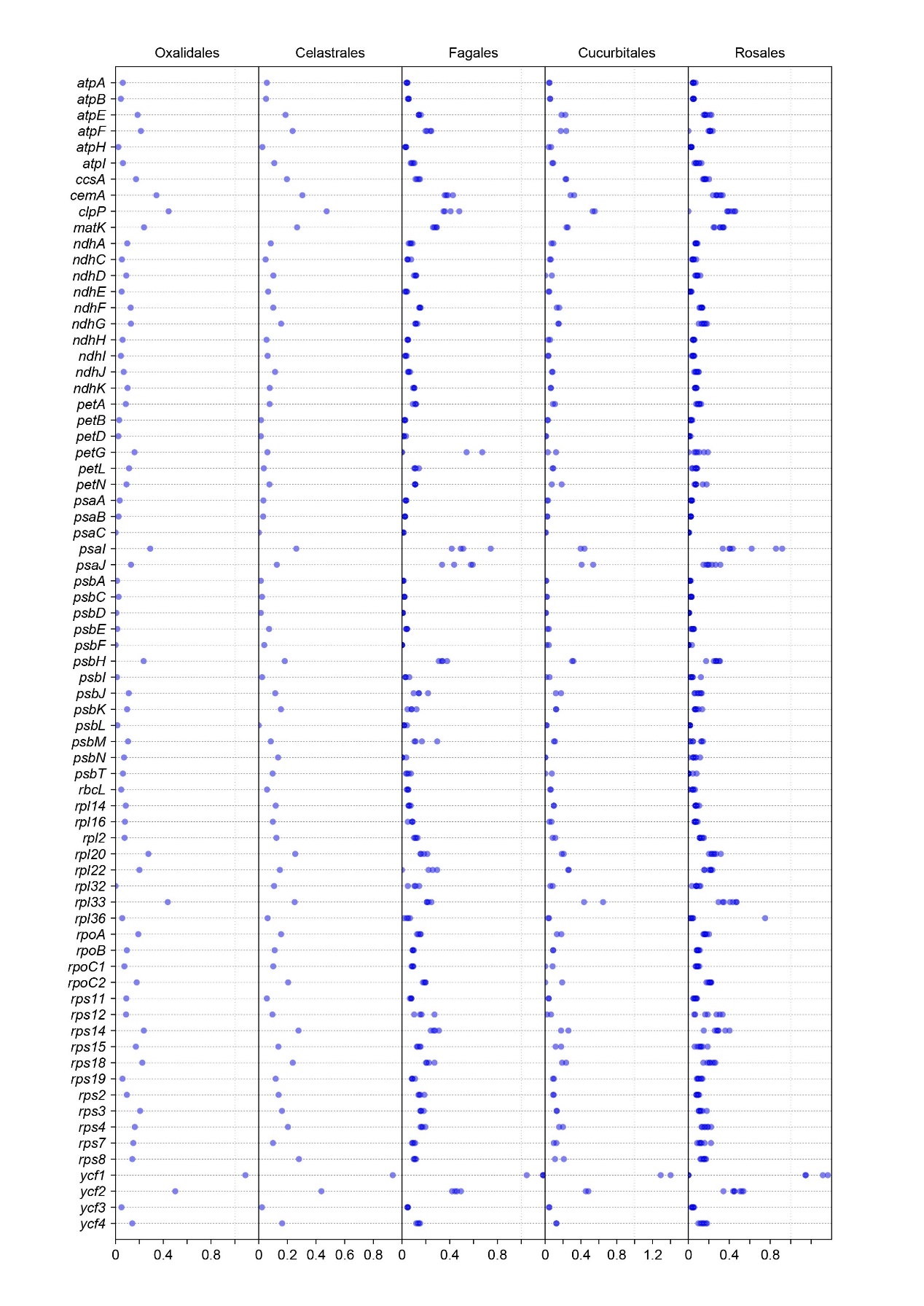


**Figure S9.** The Ka/Ks values of chloroplast protein-coding genes in species from Oxalidales, Celastrales, Fagales, Cucurbitales and Rosales (Rosid I clade).


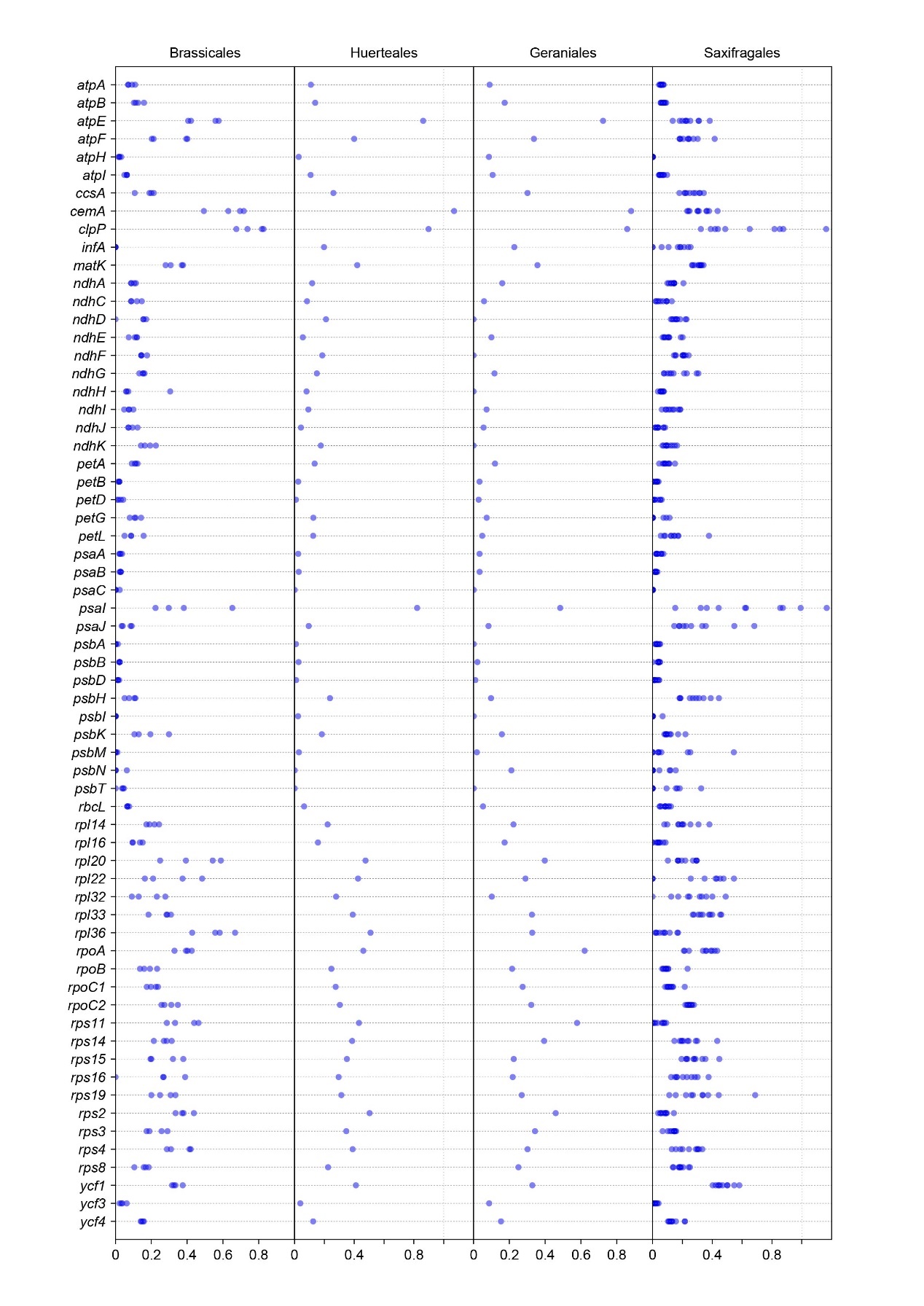


**Figure S10.** The Ka/Ks values of chloroplast protein-coding genes in species from Brassicales (Rosid II), Huerteales (Rosid II), Geraniales and Saxifragales.

**Table S1.** GC content in the 14 mangrove chloroplast genomes.

| **Species** | **IR** | **SSC** | **LSC** | **Total** |
| --- | --- | --- | --- | --- |
| *Pongamia pinnata* | 0.42 | 0.28 | 0.32 | 0.35 |
| *Avicennia marina* | 0.43 | 0.33 | 0.37 | 0.39 |
| *Excoecaria agallocha* | 0.42 | 0.29 | 0.33 | 0.36 |
| *Bruguiera sexangula* | 0.42 | 0.29 | 0.33 | 0.35 |
| *Kandelia obovata* | 0.42 | 0.28 | 0.32 | 0.35 |
| *Rhizophora stylosa* | 0.42 | 0.29 | 0.32 | 0.35 |
| *Ceriops tagal* | 0.42 | 0.29 | 0.33 | 0.35 |
| *Hibiscus tiliaceus* | 0.43 | 0.31 | 0.35 | 0.37 |
| *Heritiera littoralis* | 0.43 | 0.32 | 0.35 | 0.37 |
| *Thespesia populnea* | 0.43 | 0.32 | 0.35 | 0.37 |
| *Sonneratia ovata* | 0.43 | 0.31 | 0.35 | 0.37 |
| *Laguncularia racemosa* | 0.43 | 0.30 | 0.35 | 0.37 |
| *Pemphis acidula* | 0.43 | 0.30 | 0.34 | 0.36 |
| *Xylocarpus moluccensis* | 0.43 | 0.32 | 0.36 | 0.38 |

**Table S2**. The 57 public terrestrial species used for genomic comparison analyses (SSR, phylogeny and evolution)

| **Species name** | **Family** | **Order** | **NCBI accession** |
| --- | --- | --- | --- |
| *Echinacanthus lofouensis* | Acanthaceae | Lamiales | NC_035876.1 |
| *Bretschneidera sinensis* | Akaniaceae | Brassicales | NC_037753.1 |
| *Liquidambar formosana* | Altingiaceae | Saxifragales | NC_023092.1 |
| *Spondias bahiensis* | Anacardiaceae | Sapindales | NC_030526.1 |
| *Ostrya rehderiana* | Betulaceae | Fagales | NC_028349.1 |
| *Arabis alpina* | Brassicaceae | Brassicales | NC_023367.1 |
| *Boswellia sacra* | Burseraceae | Sapindales | NC_029420.1 |
| *Humulus lupulus* | Cannabaceae | Rosales | NC_028032.1 |
| *Carica papaya* | Caricaceae | Brassicales | NC_010323.1 |
| *Euonymus japonicus* | Celastraceae | Celastrales | NC_028067.1 |
| *Cercidiphyllum japonicum* | Cercidiphyllaceae | Saxifragales | NC_037940.1 |
| *Hirtella racemosa* | Chrysobalanaceae | Malpighiales | NC_024060.1 |
| *Tarenaya hassleriana* | Cleomaceae | Brassicales | NC_034364.1 |
| *Garcinia mangostana* | Clusiaceae | Malpighiales | NC_036341.1 |
| *Corynocarpus laevigatus* | Corynocarpaceae | Cucurbitales | NC_014807.1 |
| *Sedum sarmentosum* | Crassulaceae | Saxifragales | NC_023085.1 |
| *Coccinia grandis* | Cucurbitaceae | Cucurbitales | NC_031834.1 |
| *Daphniphyllum oldhamii* | Daphniphyllaceae | Saxifragales | NC_037883.1 |
| *Elaeagnus macrophylla* | Elaeagnaceae | Rosales | NC_028066.1 |
| *Erythroxylum novogranatense* | Erythroxylaceae | Malpighiales | NC_030601.1 |
| *Ricinus communis* | Euphorbiaceae | Malpighiales | NC_016736.1 |
| *Glycine soja* | Fabaceae | Fabales | NC_022868.1 |
| *Castanopsis echinocarpa* | Fagaceae | Fagales | NC_023801.1 |
| *Melianthus villosus* | Francoaceae | Geraniales | NC_023256.1 |
| *Hypseocharis bilobata* | Geraniaceae | Geraniales | NC_023260.1 |
| *Myriophyllum spicatum* | Haloragaceae | Saxifragales | NC_037885.1 |
| *Sinowilsonia henryi* | Hamamelidaceae | Saxifragales | NC_036069.1 |
| *Itea chinensis* | Iteaceae | Saxifragales | NC_037884.1 |
| *Juglans regia* | Juglandaceae | Fagales | NC_028617.1 |
| *Linum usitatissimum* | Linaceae | Malpighiales | NC_036356.1 |
| *Lagerstroemia fauriei* | Lythraceae | Myrtales | NC_029808.1 |
| *Byrsonima coccolobifolia* | Malpighiaceae | Malpighiales | NC_037191.1 |
| *Gossypium anomalum* | Malvaceae | Malvales | NC_023213.1 |
| *Allomaieta villosa* | Melastomataceae | Myrtales | NC_031875.1 |
| *Azadirachta indica* | Meliaceae | Sapindales | NC_023792.1 |
| *Morus notabilis* | Moraceae | Rosales | NC_027110.1 |
| *Morella rubra* | Myricaceae | Fagales | NC_035006.1 |
| *Eucalyptus obliqua* | Myrtaceae | Myrtales | NC_022378.1 |
| *Oenothera grandiflora* | Onagraceae | Myrtales | NC_029211.1 |
| *Averrhoa carambola* | Oxalidaceae | Oxalidales | NC_033350.1 |
| *Paeonia obovata* | Paeoniaceae | Saxifragales | NC_026076.1 |
| *Passiflora edulis* | Passifloraceae | Malpighiales | NC_034285.1 |
| *Penthorum chinense* | Penthoraceae | Saxifragales | NC_023086.1 |
| *Ziziphus jujuba* | Rhamnaceae | Rosales | NC_030299.1 |
| *Prinsepia utilis* | Rosaceae | Rosales | NC_021455.1 |
| *Citrus aurantiifolia* | Rutaceae | Sapindales | NC_024929.1 |
| *Salix interior* | Salicaceae | Malpighiales | NC_024681.1 |
| *Sapindus mukorossi* | Sapindaceae | Sapindales | NC_025554.1 |
| *Mukdenia rossii* | Saxifragaceae | Saxifragales | NC_037495.1 |
| *Leitneria floridana* | Simaroubaceae | Sapindales | NC_030482.1 |
| *Tapiscia sinensis* | Tapisciaceae | Huerteales | NC_036960.1 |
| *Aquilaria sinensis* | Thymelaeaceae | Malvales | NC_029243.1 |
| *Ulmus davidiana* | Ulmaceae | Rosales | NC_032718.1 |
| *Boehmeria spicata* | Urticaceae | Rosales | NC_036989.1 |
| *Viola seoulensis* | Violaceae | Malpighiales | NC_026986.1 |
| *Vitis rotundifolia* | Vitaceae | Vitales | NC_023790.1 |
| *Larrea tridentata* | Zygophyllaceae | Zygophyllales | NC_028023.1 |

**Table S3.** The assessment and comparison of phylogenetic trees.

| Consel * | | | | | | | | | | |
| --- | --- | --- | --- | --- | --- | --- | --- | --- | --- | --- |
| rank | Item ^#^ | **obs** | **au** | np | bp | pp | kh | sh | wkh | wsh |
| 1 | 4 | 0 | 0.629 | 0.225 | 0.06 | 0.269 | 0.505 | 0.983 | 0.505 | 0.984 |
| 2 | 2 | 0 | 0.628 | 0.225 | 0.085 | 0.269 | 0.495 | 0.971 | 0.495 | 0.971 |
| 3 | 1 | 0 | 0.627 | 0.225 | 0.073 | 0.269 | 0.413 | 0.962 | 0.413 | 0.947 |
| 4 | 3 | 1.6 | 0.444 | 0.217 | 0.012 | 0.052 | 0.38 | 0.84 | 0.148 | 0.755 |
| 5 | 6 | 71.8 | 0.001 | 3.00E-04 | 0.001 | 2.00E-32 | 0.002 | 0.004 | 0.001 | 0.005 |
| 6 | 5 | 80.8 | 5.00E-04 | 1.00E-04 | 1.00E-04 | 2.00E-36 | 0.001 | 0.001 | 4.00E-04 | 0.002 |

* rank, the descending order of confidence of the trees; item, the label for the tree; obs, the observed log-likelihood difference; au, the p-value of the approximately unbiased test calculated from the multiscale bootstrap; np, the bootstrap probability calculated from the multiscale bootstrap; bp, the bootstrap probability calculated in the usual manner; pp, bayesian posterior probability calculated by the BIC approximation. kh, the Kishino-Hasegawa test; sh, the Shimodaira-Hasegawa test; wkh, the weighted Kishino-Hasegawa test; wsh, the weighted Shimodaira-Hasegawa test

**#Items:** the labels of the numbered trees are:

1: BI tree using whole gene set, Figure 1

2: ML tree using whole gene set, Figure S3

3: BI tree using whole gene set with partition model, Figure S4

4: ML tree using whole gene set with partition model, Figure S5

5: BI tree using conserved 4 genes, Figure S6

6: ML tree using conserved 4 genes, Figure S7
